# Flux analysis of inborn errors of metabolism

**DOI:** 10.1007/s10545-017-0124-5

**Published:** 2018-01-09

**Authors:** D.-J. Reijngoud

**Affiliations:** 1 Section of Systems Medicine and Metabolic Signaling, Laboratory of Pediatrics, Department of Pediatrics, University Medical Center Groningen, University of Groningen, Groningen, The Netherlands; 2 Center of Liver, Digestive and Metabolic Diseases, University Medical Center Groningen, University of Groningen, Groningen, The Netherlands; 3 European Research Institute of the Biology of Ageing, Internal ZIP code EA12, A. Deusinglaan 1, 9713 AV Groningen, The Netherlands

## Abstract

Patients with an inborn error of metabolism (IEM) are deficient of an enzyme involved in metabolism, and as a consequence metabolism reprograms itself to reach a new steady state. This new steady state underlies the clinical phenotype associated with the deficiency. Hence, we need to know the flux of metabolites through the different metabolic pathways in this new steady state of the reprogrammed metabolism. Stable isotope technology is best suited to study this. In this review the progress made in characterizing the altered metabolism will be presented. Studies done in patients to estimate the residual flux through the metabolic pathway affected by enzyme deficiencies will be discussed. After this, studies done in model systems will be reviewed. The focus will be on glycogen storage disease type I, medium-chain acyl-CoA dehydrogenase deficiency, propionic and methylmalonic aciduria, urea cycle defects, phenylketonuria, and combined D,L-2-hydroxyglutaric aciduria. Finally, new developments are discussed, which allow the tracing of metabolic reprogramming in IEM on a genome-wide scale. In conclusion, the outlook for flux analysis of metabolic derangement in IEMs looks promising.

## Introduction

Inborn errors of metabolism (IEMs) form a large class of genetic diseases involving congenital disorders of metabolism. The majority are due to defects of single genes that code for enzymes that facilitate conversion of substrates into products. Although individual IEMs are rare, collectively they represent a large and diverse group of diseases. The majority of IEMs has a prevalence of less than 10 per 100,000 births with a total prevalence of all IEMs reaching 40 per 100,000 births, although the prevalence varies considerably worldwide based on the racial and ethnic composition of the population. From a societal point of view IEMs are an important burden of the medical care systems in Western countries where the most common communicable diseases are eradicated.

Although several approaches are used to treat patients with an IEM, they all have the common goal to overcome the biochemical aberrations caused by the primary enzyme defect. Therefore, understanding of the molecular and biochemical etiologies of the many IEMs is essential for successful treatment of these patients. Flux analyses by stable isotope methodology forms an important modality in the study of IEMs.

When an enzyme is deficient, metabolism seeks a new steady state, away from its physiological homeostasis. A new balance of fluxes associated with the consumption and production of metabolites characterizes the new steady state. A flux is the actual flow of metabolites through a metabolic pathway and is determined by both the rate of supply of substrates and the capacity of that pathway to convert them into products. Hence, neither changes in concentrations of intermediates nor changes in enzyme concentrations and/or activities of the reprogrammed metabolism separately hold information on the changes of the distribution of fluxes. The question arises of how to measure these changes. This review focuses mainly on studies applying stable isotope technology with mass spectrometric detection of mass isotopomer distributions (MID) of metabolites after separation by chromatography. In the next section, a short overview of the relevant stable isotope technology will be given followed by a discussion of the experimental studies over the last decades of a selected group of IEM; glycogen storage disease type I (GSD I), medium-chain acyl-CoA dehydrogenase (MCAD) deficiency, propionic aciduria (PA) and methylmalonic aciduria (MMA), urea cycle defects (UCD), phenylketonuria (PKU) and combined D,L-2-hydroxyglutaric aciduria. An earlier discussion of the application of stable isotopes in IEM can be found in Eur J Pediatr 156 Supplement 1 (1997).

## Methods of stable isotope tracer technology used in studies of flux analysis in IEM

### Isotope dilution of an exogenous infused metabolite

Two applications of stable isotope tracer studies have been used most often in the field of inborn errors of metabolism, isotope dilution of an exogenous infused labeled metabolite (this section) and isotope dilution of an endogenous produced metabolite (next section). For our discussion of the application of stable isotopes two assumptions will be essential: (i) metabolism of labeled tracer and unlabeled tracee is indistinguishable and (ii) metabolism of tracee and tracer is at steady state. At steady-state no changes occur in the concentrations of tracer and tracee. Hence, rate equations reduce to algebraic equations, which can be solved easily. Moreover, at steady state the rate of production of the metabolite of interest equals the rate of its consumption. When the aim of a study is to estimate the rate of appearance of a metabolite (R_a_) in vivo in animals or humans, an experiment can be performed in which a solution of an isotopically labeled compound (tracer) is intravenously infused and the dilution of the tracer by the tracee is measured in the circulation. To keep the discussion clear, the estimation of the rate of appearance of glucose into the systemic circulation will serve as an illustration. A solution of stable isotope labeled glucose is continuously infused intravenously at a rate V_inf_ into the systemic circulation of the animal or human. Suppose glucose is labeled at all six carbon atoms with ^13^C (a.k.a. uniformly labeled glucose, [U-^13^C]-glucose). At different time points blood samples are drawn and after work-up of the samples the MID of glucose at the various time points is measured by gas chromatography coupled to mass spectrometry. Only the time points at isotopic steady-state will be used. Next the measured intensities are normalized to the sum of the intensities of all mass isotopomers comprising the measured MID and the relative contributions are calculated for glucose molecules labeled with no ^13^C atom (m_0_), 1 ^13^C atom (m_1_), 2 ^13^C atoms (m_2_) till 6 ^13^C atoms (m_6_) with ${\sum}_{\mathrm{i}=1}^6{\mathrm{m}}_{\mathrm{i}}=1$. Carbon is naturally enriched with ^13^C (about 1.07 % (Meija et al 2016)); therefore, the recorded MID contains glucose molecules labeled by natural enrichment and by the infused [U-^13^C]-glucose. After correction of the recorded MID for natural enrichment of ^13^C (Midani et al 2017) the corrected MID (M_0_ – M_6_ with ${\sum}_{\mathrm{i}=1}^6{\mathrm{M}}_{\mathrm{i}}=1$) is solely determined by the dilution of labeled glucose (${M}_6^{glc}$*(infuse)*) by unlabeled glucose entering the systemic circulation. Since measurements at steady-state are used, R_a_ can be calculated in two steps; (i) the total rate of appearance (R_a_(tot)) of glucose is calculated by Eq.1 and (ii) R_a_(tot) is corrected for the rate of infusion of labeled glucose (V_inf_) according to Eq.2 to obtain the rate of appearance (R_a_) of unlabeled glucose:

1 $$ {\mathrm{R}}_{\mathrm{a}}^{\mathrm{glc}}\left(\mathrm{tot}\right)=\frac{{\mathrm{M}}_6^{\mathrm{glc}}\left(\mathrm{infuse}\right)}{{\mathrm{M}}_6^{\mathrm{glc}}\left(\mathrm{blood}\right)}\times {\mathrm{V}}_{\mathrm{inf}}, $$

with $\frac{M_6^{glc}(infuse)}{M_6^{glc}(blood)}$ is the dilution of the tracer [U-^13^C]-glucose by the tracee unlabeled glucose and

2 $$ {\mathrm{R}}_{\mathrm{a}}^{\mathrm{glc}}={\mathrm{R}}_{\mathrm{a}}^{\mathrm{glc}}\left(\mathrm{tot}\right)\hbox{-} {\mathrm{V}}_{\mathrm{inf}} $$

It should be realized that the calculated rate of appearance represents the flux through the studied pool (in our case, glucose in the systemic circulation) at the steady state studied and does not give information on the total capacity of the metabolic pathway, nor on the flux at a different steady state.

### Isotope dilution of an endogenously produced metabolite

**Fig. 1:**
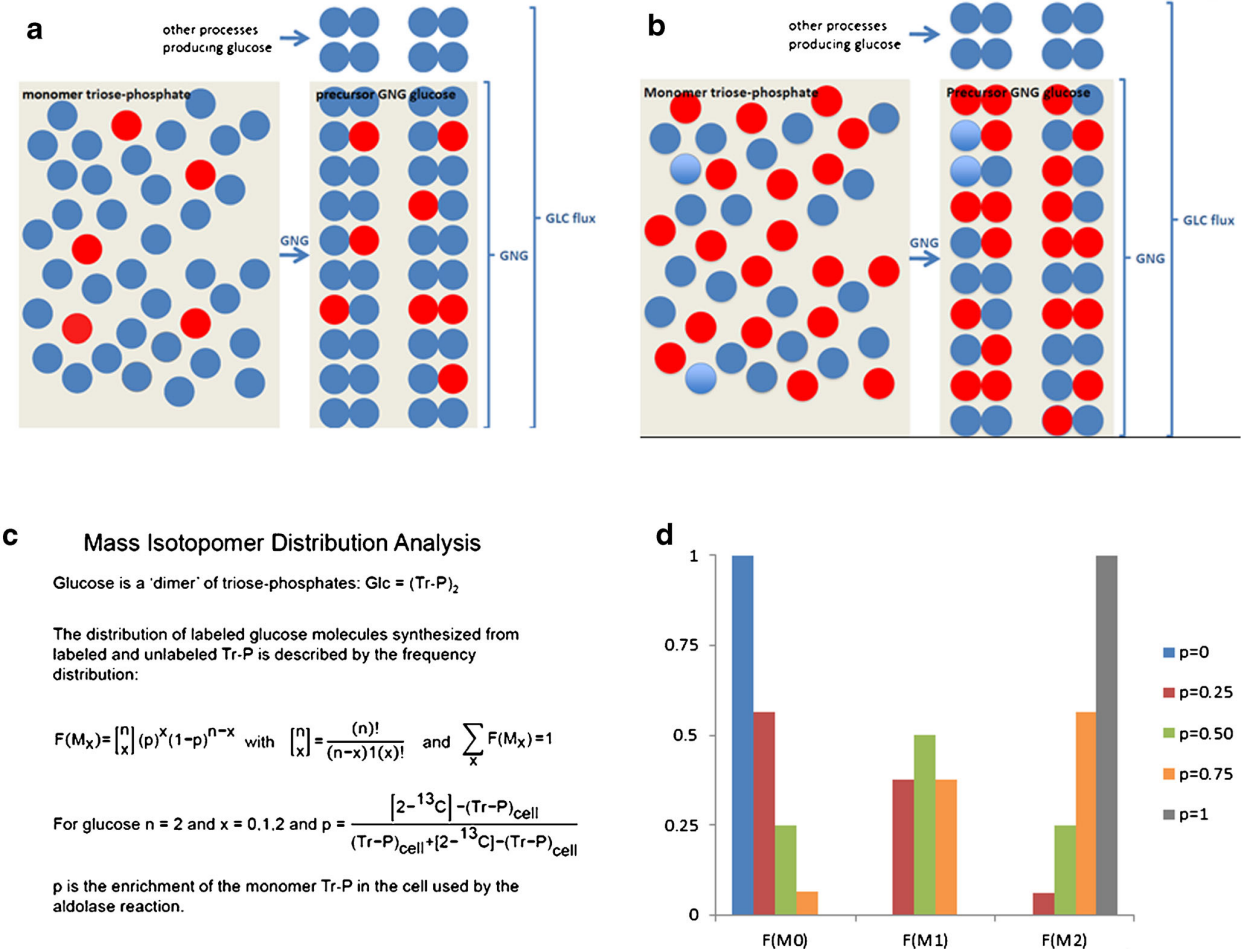
Schematic representation of ‘dimerization’ of triose-phosphates in the gluconeogenic synthesis of glucose. **a** Dimerization of triose-phosphates at low enrichment of triose-phosphate pool; **b** Dimerization of triose-phosphates at high enrichment of triose-phosphate pool; **c** The equations used to calculate the triose-phosphate enrichment from the observed MID of glucose and the ‘true’ MID of newly synthesized glucose; **d** The theoretical MID at different enrichments of the triose-phosphate pool. Blue circles represent unlabeled triose-phosphate molecules, red circles represent labeled triose-phosphate molecules, and connected circles represent glucose molecules, with all blue M_0_, single red M_1_, and double red M_2_ mass isotopomers

Let us now consider the appearance of glucose in the circulation during fasting with contributions of glycogenolysis and gluconeogenesis. When one of these processes can be estimated independent from the other, we gain information on how the appearance of glucose is determined by these two processes. To this purpose Hellerstein and coworkers developed an approach to estimate the rate of gluconeogenesis by a method they named mass isotopomer distribution analysis (MIDA) (Hellerstein and Neese 1992). Kelleher and coworkers developed a similar approach, which they named isotopomer spectral analysis (Kelleher and Masterson 1992; Kelleher and Nickol 2015; Tredwell and Keun 2015). During gluconeogenesis glucose is made from fructose-1,6-bisphosphate (Frc-1,6-P_2_), which is the product of the condensation of dihydroxyacetonphosphate (DHAP) and glyceraldehyde-3-phosphate (GA3P) by aldolase (Fig. 1). In vivo labeling of DHAP and GA3P is accomplished by intravenous infusion of a solution containing [2-^13^C]-glycerol and this will never result in complete labeling of the intracellular pools of DHAP and GA3P. Therefore, unlabeled DHAP and GA3P will always be present. Hence, condensation of labeled and unlabeled DHAP and GA3P randomly selected by aldolase will result in a mixture of labeled and unlabeled Frc-1,6-P_2_ and subsequently of labeled and unlabeled gluconeogenic glucose. When unlabeled DHAP condenses with unlabeled GA3P, it results in unlabeled M_0_ Frc-1,6-P_2_, unlabeled DHAP with labeled [2-^13^C]-GA3P results in single labeled M_1_ Frc-1,6-P_2_, similar to the condensation of labeled [2-^13^C]-DHAP with unlabeled GA3P and labeled [2-^13^C]-DHAP with labeled [2-^13^C]-GA3P results in double labeled M_2_ Frc-1,6-P_2_. The higher the enrichment of DHAP and GA3P, the higher will be the contribution of M_2_ Frc-1,6-P_2_ (compare Fig. 1a with Fig. 1b). When this set of unlabeled and labeled Frc-1,6-P_2_ is converted into glucose, it will be diluted by unlabeled glucose-6-phosphate (G6P) released by glycogenolysis. Thus, unlabeled glucose not only arises from gluconeogenesis because of the incomplete labeling of the monomeric pool of triose-phosphates but also from glycogenolysis. To correct for the synthesis of unlabeled glucose by gluconeogenesis, Hellerstein and coworkers treated gluconeogenesis as a kind of ‘polymerization’ reaction, with glucose as a ‘dimer’ of the monomer triose-phosphate (Hellerstein and Neese 1992) (Fig. 1c). The MID of labeled glucose can then be described by a frequency distribution of two monomers, labeled and unlabeled triose-phosphate. The mass isotopomers M_1_ and M_2_ of glucose are used to calculate the triose-phosphate enrichment. Next, this calculated triose-phosphate enrichment is used to calculate the ‘true’ MID (M_0_-M_2_) of newly synthesized glucose. This ‘true’ MID (‘tracer’) is compared to the measured MID of glucose (‘tracee’) in the circulation to calculate the degree of dilution of gluconeogenic glucose. In this way, Eq. 1 can be applied to calculate the rate of gluconeogenesis *V*_*gng*_. ${M}_6^{glc}(infuse)$ is replaced by ${M}_1^{glc}\left( gng\  glc\right)$. In general the calculated fraction of M_1_ gluconeogenic glucose (${M}_1^{glc}\left( gng\  glc\right)$) can be chosen because it shows the highest contribution to the MID of gluconeogenic glucose (unpublished observations). ${M}_6^{glc}(blood)$ is replaced by ${M}_1^{glc}(blood)$, the measured fraction of M_1_ glucose in the circulation and *V*_*inf*_ is replaced by *V*_*gng*_, the parameter to be calculated. R_a_, the rate of glucose appearance into the systemic circulation is measured separately by isotope dilution of [U-^13^C]-glucose. In this way the ‘true’ *V*_*gng*_ can be calculated. By subtraction of V_gng_ from R_a_ of glucose the rate of glycogenolysis is obtained.

Labeling of the triose-phosphate pool can also be achieved by infusing appreciable amounts of [U-^13^C]-glucose, which results in labeling of the triose-phosphate pool. Extensive metabolism of glucose by glycolysis, TCA cycle, and gluconeogenesis gives rise to M_0_ – M_3_ triose-phosphate and M_0_ – M_6_ glucose. This phenomenon is referred to as ‘scrambling’ of the glucose label.

MIDA can also be used to estimate de novo lipogenesis in vivo. During de novo synthesis of palmitate, FASN concatenates eight acetate molecules. Palmitate can thus be considered an ‘octamer’ of the monomer acetate. In in vivo experiments infusion of [2-^13^C]-acetate labels the intracellular pool of acetate used for lipogenesis only moderately, and labeled and unlabeled acetate will be incorporated into palmitate. Hence, newly synthesized palmitate consists of a mixture of labeled and unlabeled molecules. Unlabeled palmitate is a mixture from preexisting and lipogenic palmitate. Similar to the case of glucose, these two sources of unlabeled palmitate can be distinguished by MIDA. The MID of newly synthesized palmitate can be described by a frequency distribution of an octamer with two monomers, labeled and unlabeled acetate. The enrichment of acetate is calculated from the measured mass isotopomers M_1_-M_8_ of labeled palmitic acid. Next, the ‘true’ MID of newly synthesized palmitate (M_0_ – M_8_) is calculated. The measured MID (M_0_ – M_8_) of palmitate, in for instance VLDL, reflects the dilution of the newly synthesized palmitate by existing palmitate. The rate of de novo lipogenesis is estimated with Eq. 1, when the rate of appearance of palmitate into the studied pool is accessible for measurements. Otherwise, only the fraction of newly synthesized palmitate can be estimated. Oosterveer et al (2009) extended the method to calculate the degree of chain-elongation of palmitate into stearate and oleate.

In summary, in the case of a ‘polymerization’ reaction, a decrease in label incorporation into products can be due to (i) a decrease in enrichment of the monomers without a change in dilution of the newly synthesized product or (ii) a greater dilution of the newly synthesized product but with the same enrichment of the monomers. In the first case the MID of the labeled product will shift to lower mass isotopomers, in the latter case the relationship between the ^13^C–labeled product mass isotopomers (M_1_-M_i_) remains the same but the abundances of the mass isotopomers of the labeled product (M_1_-M_i_) decrease compared to the unlabeled product (M_0_).

### Oral loading tests

Oral loading tests are used to evaluate the degree of impairment of metabolism due to the deficiency of a particular enzyme in a non-invasive manner. In an oral loading test a labeled precursor is given and the degree of transfer of the label to a metabolic end-product is measured. In a breath test, an oral bolus of a ^13^C–labeled precursor is given and the enrichment of ^13^CO_2_ is measured. It is assumed that in this way the rate of oxidation of the substrate can be estimated. Similarly, when an oral bolus of ^15^NH_4_CO_3_ is given and the enrichment of [^15^N]-urea is measured it is assumed that the rate of urea synthesis in vivo can be estimated. Although simple to perform, these tests suffer from serious drawbacks. Very appreciable variation in test outcome is observed due to unknown dilution of the labeled precursor by metabolism in the studied subjects and by bacterial metabolism in the intestinal tract, unknown dilution of the end product, particularly when ^13^CO_2_ is used (Veeneman et al 2005), incomplete recovery of the isotope given and poor repeatability of tests (Kalivianakis et al 1997). It is for these reasons that we concluded that oral loading tests with label transfer into metabolic end products are of limited use for the quantitative assessment of the flux through the metabolic pathway under study (Reijngoud and Verkade 2017). Therefore, the oral loading test will not be discussed.

Irrespective of this conclusion, an interesting approach might be the use of [1-^13^C]-acetate to measure the residual activity of the urea cycle in patients with a urea cycle defect (Yudkoff et al 1998). Besides the appearance of ^13^C in urea, these authors also measured the enrichment of ^13^CO_2_ in exhaled air. When the changes of the enrichment of ^13^C in urea were corrected for the changes of the enrichment of breath ^13^CO_2_, the authors were able to calculate a urea cycle activity in a control subject not different from the values obtained by a primed-continuous infusion protocol (see Urea cycle defects section for discussion).

## Experimental studies

### Glycogen storage disease type I

#### Residual glucose production in patients with GSD Ia in vivo

**Fig. 2:**
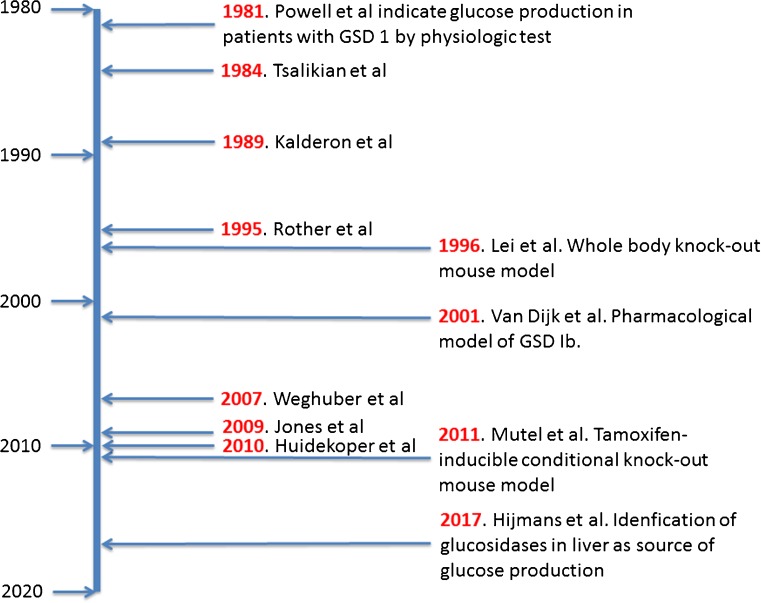
Timeline of measurements of endogenous glucose production in GSD I patients and development of model systems in mice. The references mentioned are (Powell et al 1981), (Tsalikian et al 1984), (Kalderon et al 1989a), (Rother and Schwenk 1995), (Lei et al 1996), (van Dijk et al 2001), (Weghuber et al 2007), (Jones et al 2009), (Huidekoper et al 2010), (Mutel et al 2011) (Hijmans et al 2017)

The measurement of glucose production in patients GSD I (MIM# 607008) has been pursued over many decades (see Fig. 2 for a timeline) by isotope dilution of various stable isotope labeled glucose molecules. In GSD I the conversion of glucose-6-phosphate (G6P) into glucose by the glucose-6-phosphatase complex (G6Pase) (EC# 1.3.8.7) is deficient. Hence, G6P generated by gluconeogenesis and glycogenolysis cannot be converted into glucose. This prevents gluconeogenic organs, mainly liver, to produce glucose when the prandial release of glucose by the intestine dwindles down and finally stops. One expects that in these patients no endogenous glucose production could exist. Quite to the contrary, several studies applying dilution of infused stable isotope labeled glucose have shown that in these patients an appreciable glucose production exists. The measurement of the rate of appearance of glucose in GSD Ia patients started in 1984 with a study by Tsalikian et al (1984). Five children with GSD I were infused with [6,6-^2^H_2_]-glucose intravenously and the isotope dilution of [6,6-^2^H_2_]-glucose in blood samples drawn at isotopic steady state was measured. The endogenous glucose production equaled 21.4 ± 0.2 μmol.kg^−1^.min^−1^, considerably less than in unaffected children (34.6 ± 0.2 μmol.kg^−1^.min^−1^), but still appreciable (~60% of control). They also measured the endogenous glucose production while the infusion rate of exogenous unlabeled glucose was increased stepwise. At the highest exogenous glucose infusion rate of 33.3 μmol.kg^−1^.min^−1^ endogenous glucose production could still be measured, although the rate was decreased to 7.1 ± 0.4 μmol.kg^−1^.min^−1^. Comparable observations were made by Wengler et al (2007) and Huidekoper et al (Huidekoper et al 2010). Kalderon and coworkers (Kalderon et al 1989a, Kalderon et al 1988, Kalderon et al 1989b) studied glucose production with [^13^C_6_]-glucose and measure isotope dilution and ‘scrambling’ of ^13^C–labeling in glucose by NMR. They also showed that GSD I patients produced glucose, and that the rate was lower than in healthy subjects. However, in contrast to healthy subjects, essentially no ‘scrambling’ of [U-^13^C]-glucose was observed. Jones et al (2009) measured gluconeogenesis and anaplerosis of the TCA cycle in five fed healthy subjects and five GSD Ia patients on cornstarch therapy. All subjects received a dose of acetaminophen, phenylbutyrate, and [U-^13^C]-glycerol. Blood and urine samples were collected and label distributions were measured by NMR of glucose in plasma and of the glucuronate residue of acetaminophen glucoronate, reflecting G6P labeling, and of the glutamine residue in N-phenylacetylglutamine, reflecting 2-ketoglutarate labeling. In healthy subjects 35 ± 14% of plasma glucose originated from hepatic G6P, while in patients no detectable contribution of G6P to plasma glucose was observed. Moreover, in GSD1a patients, labeling pattern in the glutamine residue of N-phenylacetylglutamine pointed to a redirection of the fluxes through the Krebs cycle to dispose of carbon normally used in gluconeogenesis to lactate production.

Collectively, these studies proved the existence of an endogenous glucose production in children with GSD I that even responded to exogenous glucose infusion. A process fundamentally different from gluconeogenesis, however, sustains the endogenous glucose production in GSD I patients. Moreover, metabolism of pyruvate was reprogrammed with a shift from anaplerosis to lactate production.

#### Glucose metabolism in animal models of GSD Ia and Ib

**Fig. 3:**
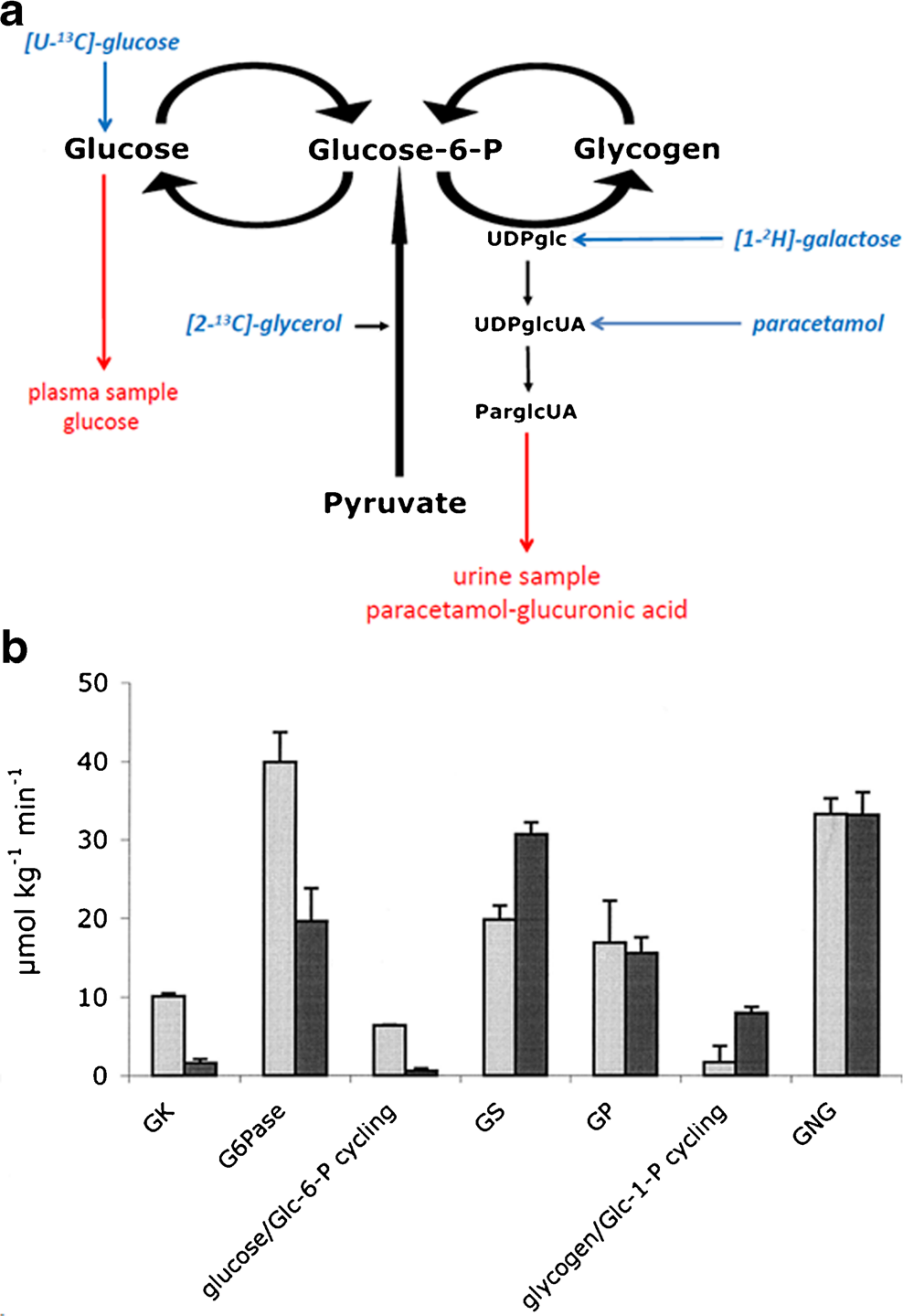
Effects of S4048 treatment in fasted rats on the fluxes through hepatic carbohydrate pathways. **a** Schematic representation of the major metabolic pathways considered in the model of hepatic carbohydrate metabolism of Hellerstein et al (1997). The pathways included are: gluconeogenesis (de novo synthesis of G6P from pyruvate); glycogenolysis; G6Pase; glucosekinase; and glycogenesis. Infused labeled metabolites are presented in blue, sampling sites and the sampled compounds are presented in red. S4048 inhibits G6P to glucose pathway. **b** The values obtained for the fluxes through the pathways depicted in (**a**) in vehicle-treated (gray bars) and S4048 treated rats (black bars). (*n* = 3) Source: (van Dijk et al 2001), *reproduced with permission from the American Society of Biochemistry and Molecular Biology*

Tsalikian et al (1984) hypothesized that glucose production in GSD Ia could be driven by the glucosidase activity of debranching enzyme or lysosomal α-glucosidase after autophagy of glycogen (a.k.a. glycophagy, (Jiang et al 2011)). These hypotheses are difficult to reconcile with the intracellular presence of glucokinase/hexokinase, which will most likely rapidly convert intracellularly released glucose into G6P. The question arises, however, of whether hepatic metabolism of G6P in GSD I is still comparable to that in healthy controls. Van Dijk et al (2001) published a series of experiments on glucose metabolism in rats acutely treated with S4048, an inhibitor of G6P transporter, mimicking GSD Ib (Fig. 3). They used the isotopic model of Hellerstein (Hellerstein et al 1997) to study the rearrangement of the flux distribution around G6P during an infusion of S4048 (Fig. 3a). Conscious and freely moving rats were infused with a multi tracer solution of [U-^13^C]-glucose, [2-^13^C]-glycerol, [1-^2^H]-galactose, acetaminophen, and when necessary S4048. During infusion blood samples were drawn and timed urine samples were collected. Label distributions were measured in glucose in plasma and the glucuronide moiety of acetaminophen glucuronic acid in urine. The label distribution in the glucuronide moiety of acetaminophen-glucuronate reflects the label distribution in the glucose moiety of UDP-glucose, which is in rapid equilibrium with G6P. In this isotope model the following fluxes are measured: (i) whole body glucose production, by dilution of infused [U-^13^C]-glucose, (ii) the glucokinase flux by the appearance of the label of [U-^13^C]-glucose in the glucuronide moiety of acetaminophen-glucuronate, (iii) gluconeogenesis, by [2-^13^C]-glycerol incorporation into glucose and glucose moiety of acetaminophen glucuronic acid, (iv) glycogen synthesis, by dilution of [1-^2^H]-galactose in the glucuronide moiety of acetaminophen glucuronic acid, (v) the G6Pase flux by the appearance of label from [1-^2^H]-galactose in glucose in plasma. Assuming flux balance at G6P all fluxes in this model can be calculated. At an infusion rate of S4048 of 0.53 ± 0.03 mg.kg^−1^.min^−1^ the G6Pase flux decreased by 50% (Fig. 3b). Intracellular concentration of G6P increased from 0.5 ± 0.1 to 2.7 ± 0.3 μmol.(g liver wet weight)^−1^. The rate of glycogenesis increased as expected in view of the increased G6P concentrations. Quite strikingly, glucokinase flux was almost abolished. It was hypothesized that the high intracellular concentrations of G6P increased fructose-6-phosphate which inhibited glucokinase activity by the stimulation of the formation of the inhibitory complex of glucokinase and glucokinase regulatory protein (see (Lenzen 2014) for a review). Hence, cytosolic glucose, released by debranching enzyme or lysosomal α-glucosidase could escape phosphorylation and be transported into the systemic circulation.

**Fig. 4:**
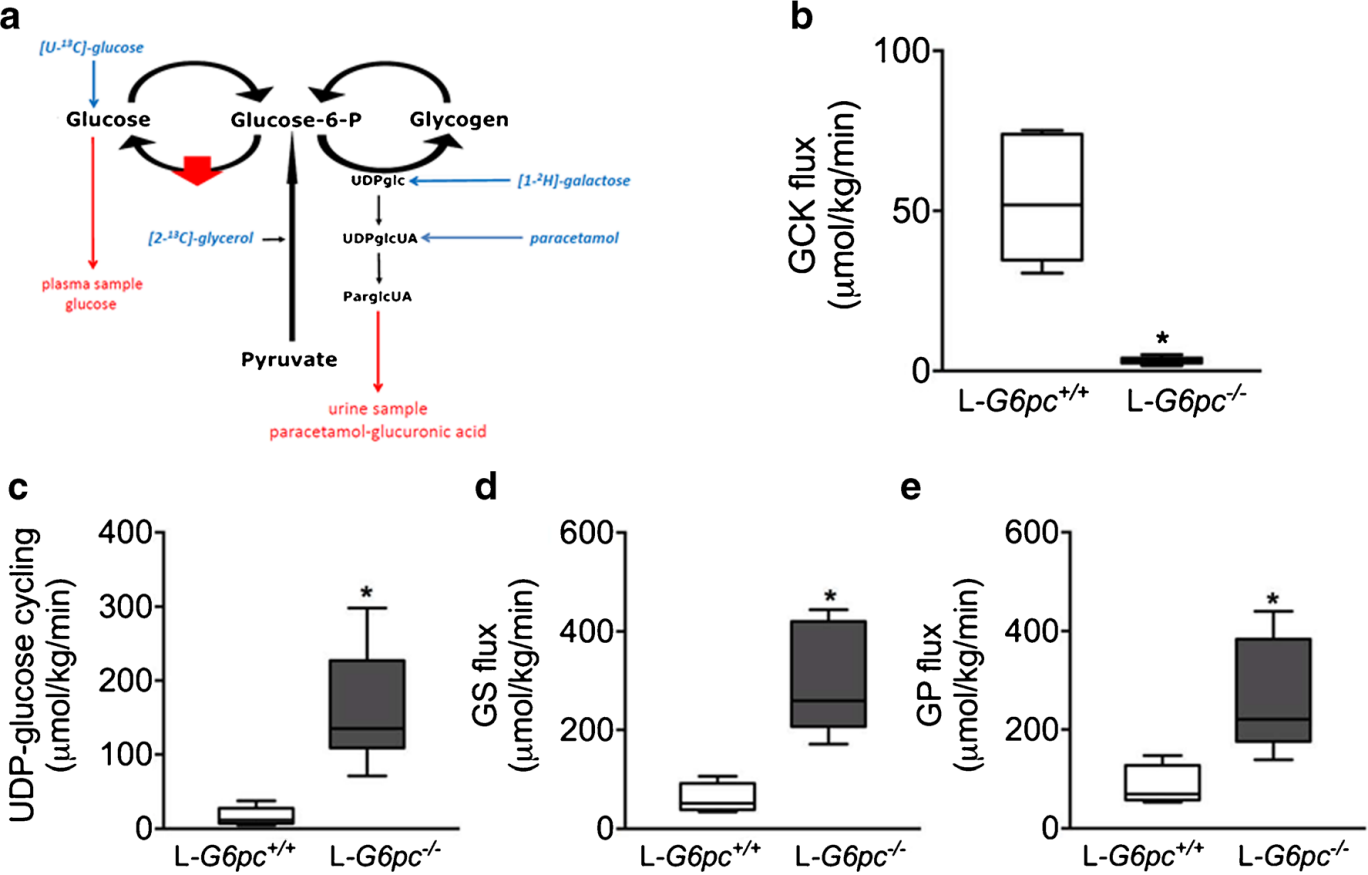
Hepatic carbohydrate metabolism in mice with a liver-specific knock-out of G6Pc. (**a**) Schematic representation of the model of hepatic carbohydrate metabolism. The red arrow indicates the liver-specific knockout of G6Pc. see legend of Fig. 3a for explanation. Figures 3(**b**), (**c**), (**d**), (**e**) show the boxplots of the flux through glucokinase, UDP-glucose cycling, glycogen synthase, and glycogen phosphorylase, respectively, with L-G6Pc^+/+^ mice (white boxes) (*n* = 6) and L-G6Pc−/− mice (black boxes) (n = 6). Values are depicted as boxplots with middle line: median, box: 25th to 75th percentiles and whiskers: 5th and 95th percentiles for n = 6 per group. *P < 0,05. Source: (Hijmans et al 2017), *reproduced with permission from the American Association of the Study of Liver Diseases*

The generation of mice with a liver specific G6Pc knockout offers new possibilities to study metabolic mechanisms more closely underlying the endogenous glucose production (Mutel et al 2011). Very recently, Hijmans et al (2017) applied the above described model of hepatic glucose metabolism to study glucose metabolism in these mice (Fig. 4a). They showed by in vivo analysis of hepatic glucose metabolism in these mice that the hepatic glucokinase flux was decreased by 95% in L-*G6pc−/−* mice (Fig. 4b). Glycogen synthase flux and phosphorylase flux were increased, resulting in an almost tenfold increase in cycling of the glucose moiety in UDP-glucose (Fig. 4b-e) in L-*G6pc−/−* mice. As a consequence, this gave rise to a tenfold increase of the release of free glucose via glycogen debranching. Furthermore, the authors showed that pharmacological inhibition of α-glucosidase activity by deoxynojirimycin almost abolished the residual glucose production in isolated *G6pc*-deficient primary hepatocytes. In conclusion, glucosidase activity sustains the glucose production in GSD Ia patients. Glucose phosphorylation is strongly suppressed. When patients with GSD Ia were treated with a glucose infusion or continuous glucose supply from cornstarch, glucose production decreased (Tsalikian et al). Hijmans et al (2017) proposed that increased exogenous glucose supply might reactivate glucokinase. Subsequently, intracellular released glucose by glucosidase activity will be phosphorylated and glucose appearance in the circulation will decrease.

#### Lipid metabolism in patients with GSD Ia in vivo

In GSD I patients lipid metabolism is severely perturbed. GSD Ia patients suffer from a combined hypertriglyceridemia and hypercholesterolemia. In a preliminary study de novo lipogenesis and cholesterol synthesis were measured in two GSD Ia patients by the infusion of [2-^13^C]-acetate, the measurement of the incorporation of ^13^C label in palmitate in VLDL and the analysis of the data by MIDA (Bandsma et al 2002). Both rates were strongly elevated. Later Bandsma et al (2008) extended these studies and included the rate of VLDL synthesis by the incorporation of infused [1-^13^C]-valine into apoB100. The results showed de novo lipogenesis was enhanced in GSD Ia patients confirming earlier results. The results of the incorporation of [1-^13^C]-valine into apoB100 showed that the rate of VLDL synthesis was not affected. In view of these results the authors hypothesized that lipolysis in GSD Ia patients must be impaired.

#### Lipid metabolism in animal model of GSD Ib

These results of lipid metabolism in GSD I patients were partially reproduced in rats treated with S4048 (Bandsma et al 2001). Acute inhibition of the G6P translocator led to a tenfold increase in de novo lipogenesis, albeit cholesterol synthesis remained unaffected. Levels of triglyceride in blood rose almost twofold during an 8 h infusion of rats with S4048, while VLDL production was not affected. Apparently, VLDL lipolysis must have been impaired. These metabolic changes were accompanied by increased expression of the genes encoding FASN and ACAT1. The enhanced expression turned out to be driven by ChREBP, a nutrient sensing-transcription factor activated by G6P, among other sugar-phosphates (Richards et al 2017). When whole body ChREBP knockout mice were treated with S4048 the increase in expression of the genes encoding FASN and ACAT1 was abolished (Grefhorst et al 2010).

In conclusion, metabolic reprogramming in GSD I resulted in an endogenous glucose production by an alternative pathway mediated by the glucosidase activity of glycogen debranching enzyme and/or lysosomal glycogen degradation. Phosphorylation of glucose was almost abolished. De novo lipogenesis and cholesterol synthesis were increased. VLDL production was not affected. Lipid levels in blood were increased, which pointed to impaired lipolysis. These changes appeared to be brought about by an active and regulated process mediated in part by G6P activated ChREBP.

### Medium-chain acyl-CoA dehydrogenase deficiency

#### Residual activity of mitochondrial medium-chain fatty acid oxidation in patients with MCAD deficiency in vivo

Heales et al measured the rate of oxidation of [1-^13^C]-octanoate in patients with MCAD (EC# 1.3.8.7) deficiency (MIM# 607008) (Heales et al 1994). They used a primed-continuous intravenous infusion of [1-^13^C]-octanoate for 4 h in four patients with MCAD deficiency after an overnight fast. The rate of appearance calculated according to Eqs. (1) and (2) ranged from 14.7 to 36.2 μmol.kg^−1^.min^−1^. Moreover, near normal values were observed for the oxidation of octanoate in these patients, varying between 6.4 and 13.1 μmol.kg^−1^.min^−1^. On average 40.0% of the infused octanoate was oxidized. The metabolic fate of the remaining 60% of the infused labeled octanoate was not reported.

It has been hypothesized that ketogenesis is impaired in patients with MCADD. Fletcher and Pitt (2001) measured the rate of ketogenesis in three well children with MCADD after 9 to 11 h fasting before starting the study period. They used a primed-continuous infusion protocol of the sodium salts of [1,3-^13^C_2_]-acetoacetate and [1,2,3,4-^13^C_4_]-3-hydroxybutyrate. They applied the two-accessible pool model to estimate the rates of ketogenesis, interconversions, and ketone utilization (Bougneres et al 1986). When compared to the rates in six healthy children (Bougneres and Ferre 1987) no differences were observed between healthy and affected children. Apparently, in these well children, ketogenesis was not impaired.

#### Animal models of mitochondrial fatty acid oxidation defects

**Fig. 5:**
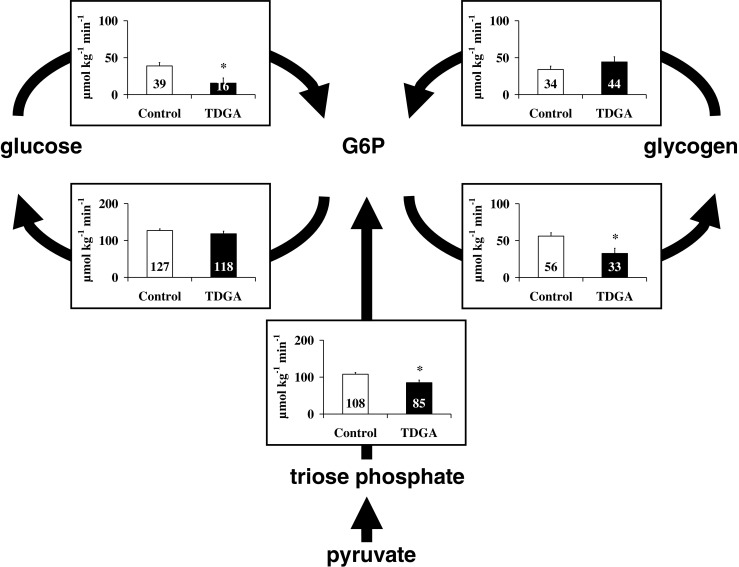
Effects of 2-tetradecylglycidic acid (TDGA) treatment on hepatic carbohydrate metabolism in mice. Mice were injected with vehicle (controls) or 30 mg/kg TDGA intraperitoneally and fasted for 9 h. Subsequently, the 6-h infusion experiment was performed. White bars show results in vehicle-treated mice, black bars in TDGA-treated mice. Data are presented as mean ± SEM, *n* = 6. *P < 0.05. Source: (Derks et al 2008b), *reproduced with permission from the American Association for the Study of Liver Disease*

Flux analysis of metabolism in various models of mitochondrial fatty acid oxidation (mFAO) defects has mainly been focused on the consequences of these defects on glucose metabolism. Patients with a defect of mFAO suffer from acute, life-threatening episodes of hypoglycemia. The acute effects of CPT-I inhibition by 2-tetradecylglycidic acid (TDGA) on glucose metabolism have been studied in mice (Fig. 5) (Derks et al 2008b). The authors applied the isotopic model of hepatic glucose metabolism and the multiple tracer infusion and sampling protocol as described above, with timed urine samples collected at hourly intervals on filter paper and without the inhibitor of the G6P transporter (Fig. 5a). Short term fasted mice received a single injection of a bolus of TDGA 9 h before the start of the experiment. At the start of the experiment, the injected mice suffer from hypoglycemia in contrast to vehicle treated mice. Changes in hepatic glucose metabolism were subtle, with a small decrease in gluconeogenesis, glycogen synthesis, and glucose phosphorylation. Irrespective of the hypoglycemia glucose production remained unaffected. In contrast, metabolic clearance rate of glucose increased considerably from ~17 ml.kg^−1^.h^−1^ to ~28 ml.kg^−1^.h^−1^. The cause of the inadequate rate of gluconeogenesis was not clear. Later studies in mice with a knockout of long chain-acylCoA dehydrogenase (Houten et al. 2013) also observed no increase in gluconeogenesis irrespective of low blood glucose concentrations in fasted mice, applying the same methodology as in the study with MCAD knockout mice. When in a separate experiment an oral bolus of alanine was given, blood glucose concentration increased transiently, indicating a possible short supply of gluconeogenic substrates. This observation has not been followed up by a stable isotope experiment to estimate gluconeogenesis in the presence of an alanine infusion. At this moment it remains an open question whether delivery of gluconeogenic substrates by peripheral tissue is hampered resulting in an inadequate rate of gluconeogenesis.

**Fig. 6:**
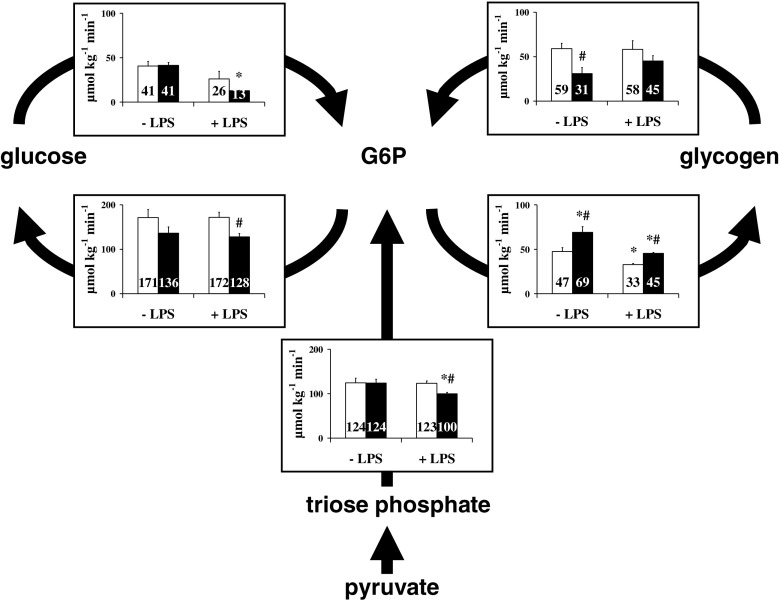
Effects of lipopolysaccharide (LPS) treatment in MCAD-deficient mice. Wild-type and MCAD−/− mice were injected with saline (-LPS) or LPS (+LPS) followed by a 9-h fast. The 6-h infusion experiment was performed subsequently. Graphs on each arrow represent the flux of the pathways as explained in Fig. 3. White bars show results in wild-type mice, black bars in MCAD–/– mice. Data are presented as the mean ± SEM, *n* = 6. ^#^*P* < 0.05 between groups. *P < 0.05 within groups. Source: (Herrema et al 2008), *reproduced with permission from the American Association for the Study of Liver Disease*

The effects of MCAD deficiency on glucose metabolism in vivo (Herrema et al 2008) were studied in mice with a deletion of MCAD (Tolwani et al 2005) by the same methods as described above. In vivo glucose metabolism was hardly affected in the mice with MCAD knocked out (Fig. 6). Only glycogen metabolism changed, while glucose production decreased slightly. Lipopolysaccharide (LPS) was used in an effort to mimic in MCAD^−/−^ mice the sudden deterioration of the clinic of MCAD deficient patients. A single bolus of LPS, to induce an acute phase response, resulted in a decreased blood glucose concentration from 6.2 ± 0.5 to 2.5 ± 0.5 mM in MCAD^−/−^ mice, not different form WT mice (8.2 ± 0.2 to 3.0 ± 0.5 mM). Glucose phosphorylation decreased more strongly after administration of LPS in MCAD^−/−^ mice compared to WT mice. Moreover, the flux through gluconeogenesis decreased in MCAD^−/−^ mice, even with the low glucose concentrations in blood.

Until now, these studies have not been able to give us detailed information on the mechanisms underlying the clinical course of patients with a mFAO defect. Recently, the group of Bakker and coworkers developed a kinetic model of the mFAO (van Eunen et al 2013, van Eunen et al 2016, Martines et al 2017). Simulations of the rate of oxidation of palmitoyl-CoA at increasing concentrations of palmitoyl-CoA showed that the ‘circular’ organization of the mFAO pathway with promiscuous enzymes resulted in a sudden and rapid decrease of the rate of the mFAO at high concentration of palmitoyl-CoA. The flux through mFAO ‘collapsed’ because the activity of medium-chain ketoacyl-CoA thiolase rapidly deteriorated upon approaching the point of ‘collapse’ (Martines et al 2017). Concomitantly, free CoASH concentration decreases to very low values because of the accumulation of acyl-CoA intermediates. When MCAD was eliminated from the model, the initial rate of palmitate oxidation did not differ from the rate calculated with MCAD present. However, the point of ‘collapse’ was reached at far lower concentrations of palmitoyl-CoA. One should be aware that very low free CoASH concentrations in mitochondria also severely interfere in pyruvate decarboxylation and TCA cycle activity. This can result in an overall impairment of the mitochondrial function. This sequence of events might take place in febrile MCADD patients upon fasting. Then metabolic demands are high and concentrations of free fatty acids in the circulation are high. In this respect it is of interest to note that MCAD can play a role in the detoxification of xenobiotic arylcarboxylates released by gut microbial metabolism. Products of microbial metabolism in the gut, like phenylpropionate and phenylbutyrate, are used as substrates in the activity assay of MCAD (Rinaldo et al 1990, Derks et al 2008a, Kormanik et al 2012). Impaired metabolism of the CoA thio-esters of arylcarboxylates might trap free CoASH in mitochondria. The point of ‘collapse’ of fatty acid oxidation will then be reached at low concentrations of long-chain acyl-CoA. Hence, when MCAD is deficient the mFAO becomes vulnerable for rapid sequestration of free CoASH already at low concentrations of fatty acid with a sudden decrease in the rate of fatty acid oxidation as a result.

Next to mitochondria, peroxisomes contribute significantly to the oxidation of long-chain fatty acids (Wanders et al 2016, Wanders 2014). The partitioning of the oxidation of fatty acids of various chain-lengths over peroxisomes and mitochondria was studied in isolated non-recirculating isolated rat heart and liver perfusion by the group of Brunengraber and coworkers (Reszko et al 2004, Bian et al 2005, Kasumov et al 2005). They studied the fate of [1-^13^C]-C_21:0_-n-, [1-^13^C]-C_18:1_-n-, [1-^13^C]-C_8:0_-n-, [3-^13^C]-C_8:0_-n-, [1-^13^C]-C_6:0_-n-, [1-^13^C]-C_4:0_-n-carboxylic acid, [1,2-^13^C_2_]-acetate and [1,12-^13^C_2_]-C_12:0_-n-dicarboxylic acid. They used the ^13^C–labeling ratio of malonyl-CoA enrichment over the enrichment of the acetate moiety of citrate to estimate the partitioning of fatty acids over peroxisomal FAO (pFAO) and mFAO. The observed ^13^C–labeling ratios indicated that, as expected, long-chain fatty acids were oxidized in mitochondria. However, concomitantly, they were vividly oxidized in peroxisomes till C_6:0_-n-carboxylic acid, followed by oxidation in mitochondria. This became clear when the ^13^C–labeling ratios were compared when [1-^13^C]-C_8:0_-n-carboxylic acid was infused with the ratio when [3-^13^C]-C_8:0_-n-carboxylic acid was infused. Only [1-^13^C]-C_8:0_-n-carboxylic acid resulted in a ^13^C–labeling ratio indicative of both pFAO and mFAO. Oxidation of [3-^13^C]-C_8:0_-n-carboxylic acid resulted in ^13^C labeling ratios indicative of mFAO. Transfer of ^13^C from [1,12-^13^C_2_]-C_12:0_-n-dicarboxylic acid was only to malonyl-CoA without labeling of the acetate moiety of citrate, indicating that oxidation started exclusively in peroxisomes. These results show that pFAO of long-chain acyl-CoA till hexanoyl-CoA contributes to the total oxidative capacity in rat liver and heart. It is not clear from the results how important pFAO is in quantative terms, since the authors did not estimate the total rate of fatty acid oxidation or the turnover of malonyl-CoA. The authors conclude that pFAO supplies acetyl-CoA for malonyl-CoA synthesis and subsequently for lipogenesis. Indeed, Oosterveer et al (2009) observed label incorporation of [2-^13^C]-acetate into palmitate indicating lipogenesis in VLDL in mice during treatment with fibrates, which stimulated mFAO and pFAO.

Wanders et al (2010) proposed fatty acid ω-oxidation (ωFAO) by the CYP4A/F subfamilies and subsequent peroxisomal oxidation of the resulting dicarboxylic acids as a rescue pathway in mFAO disorders. Jin and coworkers (Jin et al 2015) studied the partitioning of [U-^13^C]-C_12_-, [U-^13^C]-C_9_-, and [U-^13^C]-C_5_-n-dicarboxylic acids over mFAO and pFAO in perfused isolated rat livers by measuring the labeling of various acetyl-CoA proxies. [U-^13^C]-C_12:0_-n- and [U-^13^C]-C_9:0_-n-dicarboxylic acids were partially oxidized in peroxisomes with terminal oxidation in mitochondria. This is in line with the observation of label transfer of ^13^C from [1,12-^13^C_2_]-C_12:0_-n-dicarboxylic acid to malonyl-CoA catalyzed exclusively by peroxisomes. Furthermore, they observed that oxidation of [U-^13^C]-C_5:0_-n-dicarboxylic acid (glutaric acid) occurred in mitochondria without a contribution of peroxisomes. As has been noticed above, the authors did not estimate absolute oxidative rates of ωFAO of long-chain fatty acids followed by pFAO of dicarboxylic acids. So, it remains unclear how important these contributions are to the total flux of FAO.

In conclusion, in mice with an mFAO defect, the supply of gluconeogenic substrates appeared to be limited, while the capacity of gluconeogenesis seems not to be affected. Peripheral proteolysis might be too low to supply substrates in adequate amounts. The question arises whether there is a place for a protein-enriched diet in the treatment of mFAO deficiencies. The role of pFAO and ωFAO in patients with a defect in mFAO clearly warrants further study (see Violante et al 2013, Wanders et al 2016). The question arises of how important pFAO is in humans in comparison with mFAO. In this respect, the clinical trials with PPARα-ligand benzofibrate are of interest (Bonnefont et al 2009) (Gobin-Limballe et al 2007).

### Propionic and methylmalonic aciduria

#### Propionate metabolism in patients with propionic and methylmalonic aciduria

The remaining capacity to detoxify propionic acid in patients with PA (MIM# 606054) or MMA (MIM# 2510000) had been measured by Thompson and coworkers (Thompson et al 1990b) (Thompson et al 1990a). In these patients detoxification of propionate is severely hampered due to the deficiency of propionyl-CoA carboxylase (EC# 6.4.1.3) or methylmalonyl-CoA mutase (EC# 5.4.99.2). Propionic acid is the product of the oxidation of the amino acids valine, isoleucine, methionine, and threonine; but more importantly, propionic acid is one of the short-chain fatty acids (SCFAs) produced in high amounts by the gut microbiota together with acetate and butyrate (Besten et al 2014). Thompson and coworkers infused three children with PA and three children with MMA with a multi-tracer solution of [1-^13^C]-propionate, [*ring*-^5^H_2_]-phenylalanine and [1-^13^C]-tyrosine. The infusion of [*ring*-^5^H_2_]-phenylalanine and [1-^13^C]-tyrosine was used to calculate the rate of phenylalanine hydroxylation as a measure of protein catabolism and the rate of irreversible catabolism of the amino acids valine, isoleucine, methionine, and threonine. The appearance rate of propionate into the systemic circulation in the three children with PA was 0.75, 0.76, and 0.82 μmol.kg^−1^.min^−1^ and in the three children with MMA 1.92, 1.45, and 0.77 μmol.kg^−1^.min^−1^. When patients were treated with the antibiotic metronidazole to eliminate propionate production by the gut microbiota, propionate production decreased 22 ± 8%. Very recently Boets et al measured the rate of appearance in the systemic circulation of SCFA in healthy adults (Boets et al). The estimated appearance rate of propionate was 0.27 ± 0.09 μmol.kg^−1^.min^−1^ (*n* = 12) considerably less than in patients with PA and MMA. Next, Thompson and coworkers estimated the oxidation of propionate in the same patients (Thompson et al 1990b). In the children with PA, oxidation was 0.18–0.60 μmol.kg^−1^.min^−1^, and in the children with MMA, 0.25–1.08 μmol.kg^−1^.min^−1^. Lower oxidation rates were observed in healthy subjects (0.08–0.32 μmol.kg^−1^.min^−1^). These values were, however, not corrected for differences in circulating propionate concentrations.

#### Propionate metabolism in ex vivo models

**Fig. 7:**
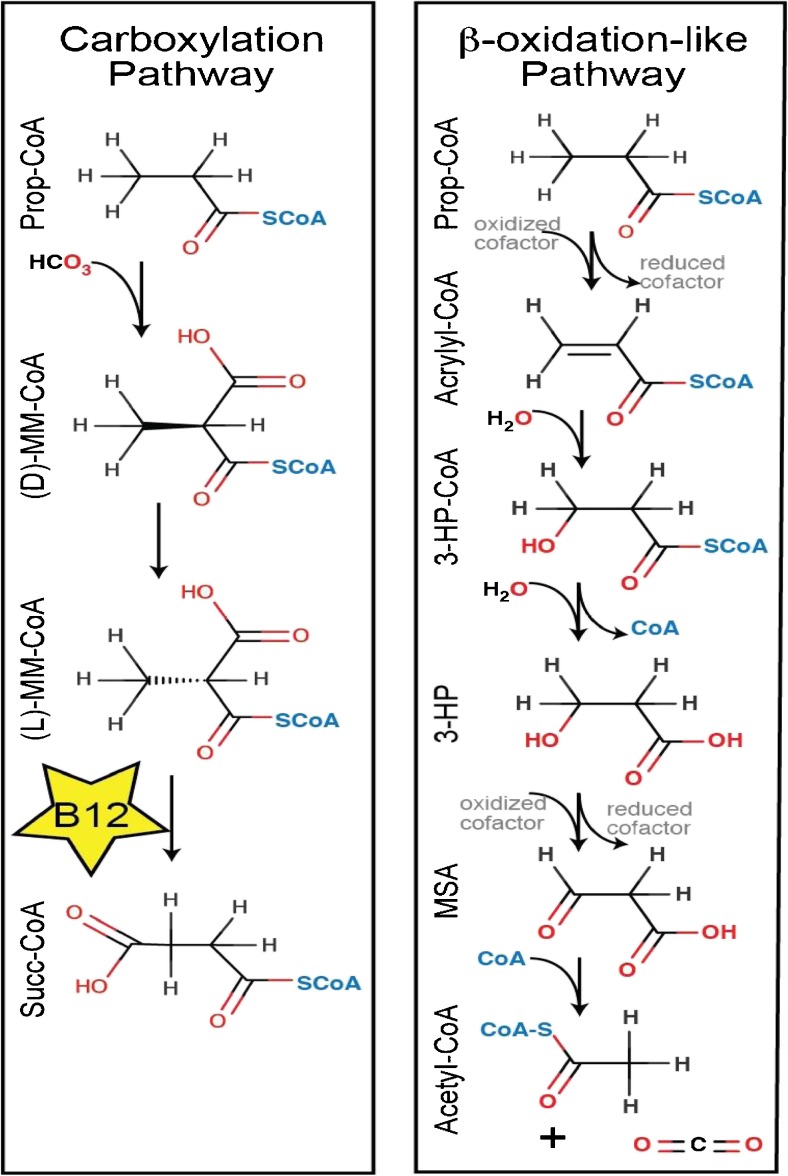
Schematic representation of the two metabolic pathways of propionate in humans. **a** The canonical vitamin B_12_ dependent pathway of propionate conversion into succinate. **b** The modified β-oxidation pathway of propionate with cytotoxic intermediates. MM-methylmalonyl, 3HP-3-hydroxypropionate, MSA-malonic semialdehyde. Source: (Watson et al 2016). *Reproduced with permission from the publisher of eLife journal*

Apparently, an alternative pathway for oxidation of propionate might be operative in patients with PA or MMA. In the 1970s Ando et al (Ando et al. 1972a, Ando et al. 1972b) alluded to the possibility of a modified β-oxidation pathway of propionate. Recent studies in *C. elegans* and HepG2 cells (Watson et al 2016) showed the existence of such an alternative oxidation pathway (Fig. 7). In humans this pathway consists of five enzymes starting at propionyl-CoA dehydrogenation by ACADSB (EC# 1.3.8.5), hydration of the product acrylyl-CoA by ECHS1 (EC# 4.2.1), after which the product 3-hydroxypropionyl-CoA is hydrolyzed by HIBCH (EC# 3.1.2.4) and 3-hydroxypropionate is formed. Further oxidation to malonic semialdehyde is catalyzed by the transhydrogenase ADHFE1 (a.k.a. HOT) (EC# 1.1.99.24), while reducing 2-ketoglutarate into D-2-hydroxyglutarate. Finally, malonic semialdehyde is oxidative decarboxylated by ALDH6A1 (EC# 1.2.1.27) into acetyl-CoA and CO_2_.

Experiments with [U-^13^C]-propionate in the presence and absence of labeled and unlabeled 3-hydropropionate by Wilson and coworkers (Wilson et al 2017) in non-recirculating perfused isolated rat liver were fully compatible with the presence of the proposed modified β-oxidation for propionate. Labeling of 3-hydroxypopionate by [U-^13^C]-propionate was almost complete. Appreciable amounts of M_2_ acetyl-CoA were observed indicative of the presence of the modified β-oxidation pathway. As expected, extensive labeling of succinate was also observed via the canonical, vitamin B_12_ dependent pathway, with M_3_ succinate originating from the incorporation of infused M_3_ [U-^13^C]-propionate and M_2_ succinate due to recycling of M_3_ succinate through the TCA cycle. The addition of 3-hydroxypropiopnate to the perfusate altered the MID of succinate profoundly. The fractional contribution of M_2_ succinate almost disappeared, pointing to a strong inhibition of the TCA cycle by 3-hydroxypropionate.

In conclusion, the observations made by Thompson and coworkers deserve to be re-investigated in light of these new findings. When one considers stimulation of the modified β oxidation pathway as a therapeutic option in patients with propionic and methylmalonic aciduria, one should realize that intermediates in this pathway are cytotoxic, in contrast to the canonical vitamin B_12_-dependent pathway via methylmalonate and succinate. Appreciable reaction rates will only be obtained at elevated concentrations of propionate such as observed in patients with propionic and methylmalonic aciduria.

### Urea cycle defects

#### Estimation of the urea synthetic flux in vivo in patients with urea cycle defects

Hepatic urea synthesis is the major pathway by which virtually all terrestrial mammals rid themselves of excess nitrogen. Major sources of nitrogen are portal ammonia derived from host urea hydrolyzed by bacterial urease in the gut, the activity of portal glutaminase, and whole-body amino acid oxidation (Veeneman et al 2004). UCDs come in a wide spectrum of clinical presentations. Hence, it becomes of importance to have a method that estimates the residual activity of the urea cycle when one of the enzymes is deficient. In vivo urea synthesis in healthy subjects and patients with a UCD has been estimated during a primed-continuous intravenous infusion of [^18^O]-urea or [^13^C]-urea and measurement of isotope dilution of labeled urea in blood. The values obtained for urea synthesis in healthy subjects varied between 3.7 ± 0.2 μmol.kg^−1^.min^−1^ (Matthews and Downey 1984), 2.2 ± 0.3 μmol.kg^−1^.min^−1^ (Lee et al 2000), and 3.6 ± 0.5 μmol.kg^−1^.min^−1^ (Scaglia et al 2003). Our measurements in adult nephrotic patients by a different, kinetic isotopic method gave a value of 3.4 ± 0.2 μmol.kg^−1^.min^−1^ (Veeneman et al 2004). Subsequently, urea synthesis in treated patients had been estimated (Lee et al 2000). In patients with a null mutation of one of the genes encoding for the enzymes of the urea cycle the rate of urea synthesis was not different from 0 μmol.kg^−1^.h^−1^ (−0.01 ± 0.03 μmol.kg^−1^.min^−1^), after correction for urea production of therapeutic arginine and citrulline supplementation. To circumvent this correction the authors used an infusion protocol with [5-^15^ N]-glutamine and measured the molar enrichment of [5-^15^ N]-glutamine and [^15^N]-urea to estimate the transfer of ^15^N from glutamine to urea. They expressed the extent of transfer as a ratio of [^15^N]-urea enrichment over [5-^15^ N]-glutamine enrichment (Scaglia et al 2002, Lee et al 2000). They observed a ratio of 0.42 ± 0.06 in healthy controls, 0.003 ± 0.007 in patients with a null mutation, and intermediate values in the other groups of urea cycle defects (0.16 ± 0.04 to 0.35 ± 0.11). No relation was observed between the severity of the disease in each of the patients individually and their value of the ratio, only groups of patients could be distinguished.

Scaglia and coworkers (Scaglia et al 2003) studied the first pass extraction of orally given ^15^NH_4_Cl in healthy subjects and in female OTC carriers. In healthy subjects 61 ± 8% of the enteral supplied ^15^N was transferred to urea by the liver on the first pass. A total of 17 ± 8% of the orally supplied ^15^N was recovered in glutamine and 22 ± 6% escaped extraction on the first pass by the liver. In female OTC carriers first pass transfer of nitrogen by the liver from orally supplied ^15^NH_4_Cl to urea decreased to 11 ± 8% and the transfer to glutamine increased to 29 ± 8%. A total of 60 ± 6% of enteral ^15^NH_4_Cl escaped the first pass. No difference was observed in the final extent of label incorporation of orally administered tracer into urea between the two groups (81 ± 4% vs 72 ± 4%, mean ± SE, healthy subject vs OCTD carriers, respectively). Next, they studied the effect of phenylbutyrate on nitrogen metabolism in healthy subjects and partial-OTC deficient female subjects. Phenylbutyrate is a drug used in patients suffering from a UCD to induce alternative pathways of nitrogen disposal. All subjects received a phenylbutyrate dose of ~5.6 g.kg^−1^.d^−1^. In control subjects urea synthesis decreased from a mean of 3.0 to 2.6 μmol.kg^−1^.min^−1^ and in partial-OTC deficient female subjects from 2.6 to 2.0 μmol.kg^−1^.min^−1^. Phenylbutyrate did not affect protein catabolism, the whole-body turnover of glutamine, leucine, phenylalanine, and tyrosine nor the hydroxylation of phenylalanine into tyrosine. The clearance of leucine increased by 61% which resulted in a decreased leucine concentration in the bloodstream.

#### Tracer-based studies of nitrogen metabolism in model systems

In the liver an enteral nitrogen load is partitioned over irreversible disposal by the urea cycle and preservation by incorporation of nitrogen into glutamine. Until now most tracer studies with stable isotopes have been performed in non-recirculating perfused isolated liver preparations without a UCD (Brosnan et al 1996, Brosnan et al 2004, Nissim et al 1996, Nissim et al 1992, Nissim et al 2003, Nissim et al 1999). In urea two N-atoms are present, one derived from NH_3_ and one from aspartate. Accordingly, MIDA was applied to analyze the ^15^N–labeling pattern of urea with [*ureido*-^15^ N]-citrulline and [2-^15^ N]-aspartate in the effluent as proxies of the immediate precursor enrichment of mitochondrial carbamoylphosphate and cytosolic aspartate, respectively. The transfer of ^15^N to urea was studied from ^15^NH_3_, [2-^15^ N]-alanine, and glutamine labeled at the amino-N ([2-^15^ N]-glutamine), the amide-N ([5-^15^ N]-glutamine) or fully labeled ([2,5-^15^ N_2_]-glutamine). Collectively, the studies pointed to the important role of mitochondrial glutamate formation via reductive amination of 2-ketoglutarate by glutamate dehydrogenase in the detoxification of ammonia by supplying aspartate-N for transamination of oxaloacetate into aspartate by glutamate. The amide-^15^ N in [5-^15^ N]-glutamine was preferentially transferred to carbamoylphosphate. Furthermore, [2-^15^ N]-alanine and ^15^NH_3_ provided both nitrogen-atoms of urea but the transfer of ^15^N via aspartate was preferred by [2-^15^ N]-alanine while the transfer via citrulline was preferred by ^15^NH_3_. Perfusion of livers with NH_4_Cl strongly increased the negative nitrogen balance of perfused isolated rat liver indicative of increased hepatic proteolysis to supply enough aspartate-N to the urea cycle. Similar conclusions were reached by Yang et al (2000) in fasted dogs. Apparently, supply of glutamate by reductive amination of 2-ketoglutarate was inadequate to deal with the high demand of aspartate-N when supply of ammonia was high. Glutamate synthesis in liver mitochondria appeared to be central to ammonia detoxification.

Recent studies by Lamers and coworkers have put the results of these studies in isolated rat liver into a whole body perspective (He et al 2010, Hakvoort et al 2017). They studied intestinal ammonia detoxification in control mice and in mice with a liver knockout of glutamine synthetase. They showed that in mice ~30% of the infused ammonia is not cleared by the liver on first pass, independent of portal ammonia concentrations ≤2 mM and an intestinal infusion of ammonia ≤166 μmol NH_4_CO_3_.kg^−1^.min^−1^. Detoxification of the part of intestinal infused ammonia absorbed by the liver (~70%) was equally partitioned over urea synthesis (~35%) and glutamine synthesis (~35%). During an intravenous infusion of NH_4_CO_3_, detoxification of systemic ammonia was almost entirely dependent on glutamine synthesis in muscle at an infusion rate ≤ 20 μmol NH_4_CO_3_.kg^−1^.min^−1^. Next, the transfer of the amide-N of glutamine to urea was studied in control mice and mice with liver and muscle knockout of glutamine synthetase using a multi tracer primed-continuous infusion protocol with [5-^15^ N]-glutamine and [^18^O,^15^N_2_]-urea and the measurement of the dilution of [5-^15^ N]-glutamine and [^18^O,^15^N_2_]-urea and the appearance of [^15^N]-urea in the circulation. The whole-body rate of appearance of newly synthesized glutamine, traced with [5-^15^ N]-glutamine, was closely correlated with the rate of transfer of amide-N of newly synthesized glutamine to urea (measured as [^15^N]-urea), without changes in total urea production, measured with [^18^O,^15^N_2_]-urea. These studies clearly point to the pivotal role of glutamine in whole body nitrogen metabolism.

Marini and coworkers (Matini et al 2005, Marini et al 2006a, Marini et al 2006b) studied urea synthesis in wild-type mice and in mice hypomophic for ornithine transcarbamoyltransferase (EC# 2.1.3.3) (OTC^spf-ash^). In normal mice they measured urea synthesis by isotope dilution of a single intravenous bolus injection of [^15^N_2_]-urea and by primed continuous intravenous infusion of the same label. The rate of urea synthesis was 56 ± 12 μmol.kg^−1^.min^−1^ an order of magnitude greater than reported in humans. Urea synthesis in OTC^spf-ash^ mice was not different from control mice (52 ± 11 μmol.kg^−1^.min^−1^). When control mice and OTC^spf-ash^ mice were fed a complete mixture of amino acids at ~200 μat N.kg^−1^.min^−1^, no difference was observed in the ability to synthesize urea (mean: 107 vs 108 μmol.kg^−1^.min^−1^, control vs OTC^spf-ash^ mice, respectively). In contrast, when OTC^spf-ash^ mice were fed an unbalanced mixture of glycine and alanine at a molar ratio of 1:2 without other amino acids, at ~200 μat N.kg^−1^.min^−1^ no increase was observed in urea synthesis (mean: 71 vs 53 μmol.kg^−1^.min^−1^, control vs OTC^spf-ash^ mice). When OTC^spf-ash^ mice fed the glycine-alanine mixture were intravenously infused with ornithine at 53 μmol.kg^−1^.min^−1^ urea synthesis normalized (mean: 75 vs 76 μmol.kg^−1^.min^−1^, control vs OTC^spf-ash^ mice). Apparently, the complete mixture of amino acids generated enough ornithine to allow OTC^spf-ash^ mice to cope with the increased nitrogen load of the complete mixture of amino acid. We can compare this outcome with results of experiments where the effect was studied of  perfusion of isolated rat liver with a solution of NH_4_Cl (see above). Liver weight decreased and the conclusion was drawn that protein catabolism was enhanced to supply adequate amounts of aspartate-N to detoxify ammonia. The experiments in OTC^spf-ash^ mice point to the importance of the supply of ornithine or of its precursor arginine when the urea cycle is ‘broken’. For some UCDs this is part of the clinical management of patients.

In conclusion, not all ammonia supplied by the gut is converted into urea upon first pass by the liver. The remainder is transferred to glutamine or enters the systemic circulation where glutamine synthesis in peripheral tissue transfers ammonia-N to glutamine. The supply of 2-ketoglutarate in liver mitochondria plays an important role to supply glutamate. Newly synthesized glutamine from glutamate is a major precursor of urea synthesis. The study of whole-body nitrogen metabolism in models of UCDs holds the key to new modalities of treatment of these patients as is already shown by the studies in OTC^spf-ash^ mice. One might hypothesize that supplying precursors of glutamine synthesis alleviates the hyperammonemia (Hakvoort et al 2017). Moreover, oral supplementation with ornithine might also alleviate or even prevent hyperammonemic periods, particularly for female OTC carriers.

### Phenylketonuria

In three studies the residual activity of phenylalanine hydroxylation was measured in PKU patients (MIM# 261600) (Thompson et al 1990b; Thompson and Halliday 1990; van Spronsen et al 1998). In PKU patients the enzyme that converts phenylalanine into tyrosine (phenylalanine 4-monooxygenase (EC 1.14.16.1)) is deficient. A multi tracer primed-continuous infusion of a solution of [*phenyl-*^2^H_5_]-phenylalanine and [1-^13^C]-tyrosine was given to PKU patients and during infusion blood samples were drawn. Isotope dilution was measured of [*phenyl-*^2^H_5_]-phenylalanine, [1-^13^C]-tyrosine, and [*phenyl-*^2^H_4_]-tyrosine, the hydroxylation product of [*phenyl-*^2^H_5_]-phenylalanine. Thompson and coworkers (Thompson and Halliday 1990, Thompson et al 1990b) observed an almost normal rate of hydroxylation of phenylalanine in PKU patients of 0.08 ± 0.04 μmol.kg^−1^.min^−1^, compared to healthy subjects 0.10 ± 0.03 μmol.kg^−1^.min^−1^. In two patients with hyperphenylalaninemia, hydroxylation of phenylalanine was 0.07 and 0.09 μmol.kg^−1^.min^−1^. No relationship was observed between the apparent rate of hydroxylation and the concentration of phenyalanine in blood during the study. Applying the same experimental protocol Van Spronsen et al came to a completely different conclusion (van Spronsen et al 1998). In PKU patients they estimated the rate of phenylalanine hydroxylation to be 0.006 ± 0.002 μmol.kg^−1^.min^−1^. Their values in two healthy subjects were 0.10 and 0.07 μmol.kg^−1^.min^−1^, fully compatible with the values observed by Thompson and coworkers. No relation was found between the hydroxylation rate and the tolerance to natural protein in the diet. The reason for the lack of correlation of phenylalanine hydroxylation and measures of clinical severity is not clear. It might be that other as yet unknown pathways exist which metabolize phenylalanine. Bacterial metabolism might have interfered with the measurements of phenylalanine metabolism. To a certain extent, this might be similar to the case of host urea, which is hydrolyzed in the intestine by bacterial urease. When [^2^H_5_]-phenylalanine is hydrolyzed by bacterial phenylalanine ammonia lyase (EC 4.3.1.24) it escapes conversion into tyrosine (Clayton 2012). In this way phenylalanine leaves the circulation by two routes, and only one is traced by the experimental set up.

### Tracer-based studies of citrate metabolism in fibroblasts of patients with combined D,L-2-hydroxyglutaric aciduria

**Fig. 8:**
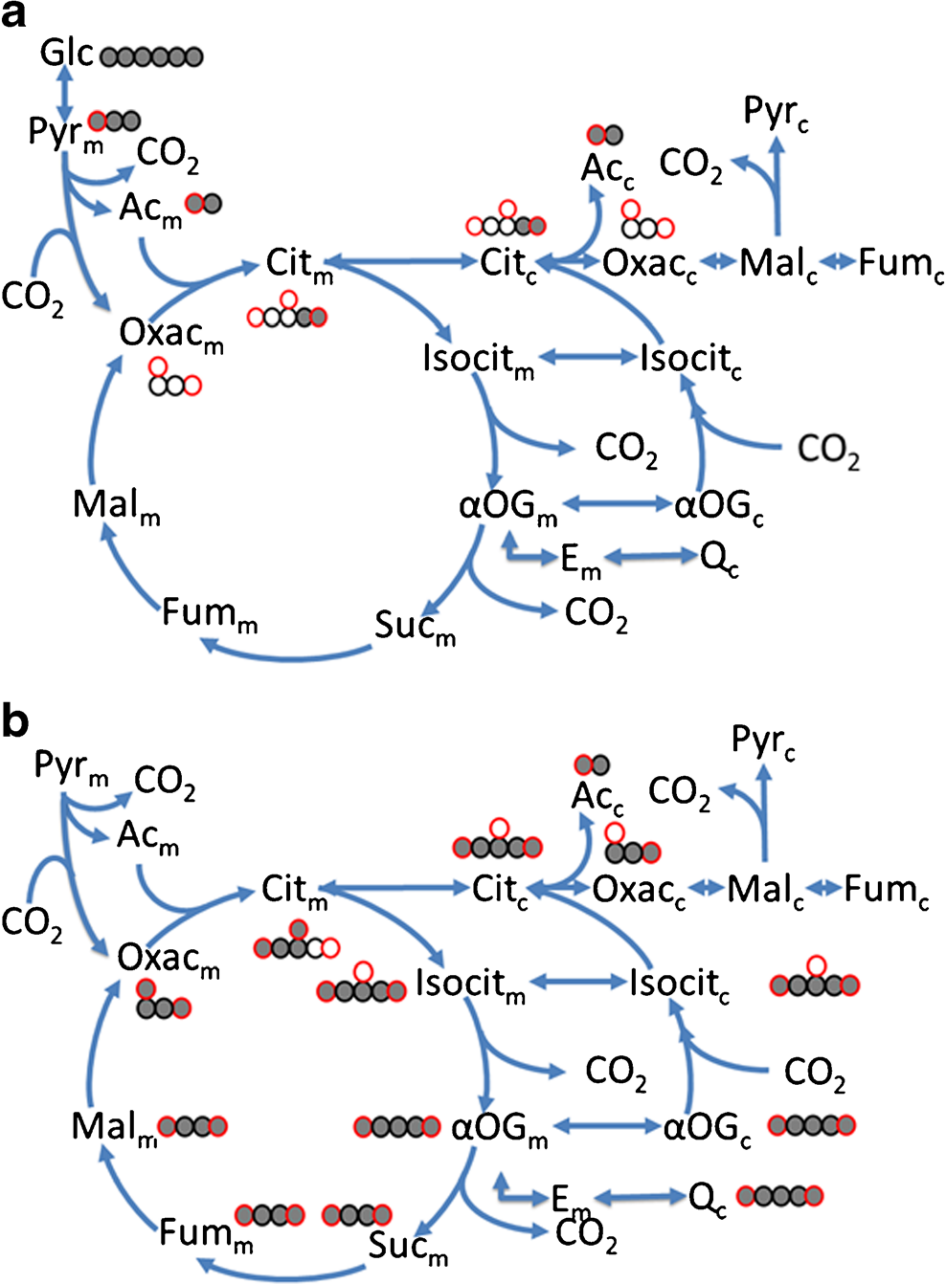
Schematic representation of TCA cycle and cytosolic metabolism of TCA intermediates. **a** Schematic representation of mitochondrial (m) and cytosolic (c) citrate metabolism and carbon atom transitions for [U-^13^C]-glucose (**b**) idem for [U-^13^C]-glutamine. Black circles represents aliphatic atoms, red circle represents carboxylic carbon atoms. Open circles represent unlabeled carbon atoms. Closed circles represent ^13^C labeled carbon atoms. Glc – glucose, Pyr – pyruvate, Ac – acetylCoA, Cit – citrate, Isocit – isocitrate, αOG – 2-ketoglutarate, Suc – succinate, Fum – fumarate, Mal – malate, Oxac – oxaloacetate, E-glutamate, Q-glutamine

The group of Struys studied metabolic reprogramming in fibroblasts of patients lacking the citrate transporter (unpublished observations). Deficiency of the mitochondrial citrate transporter (SLC25A1) results in combined D- and L-2-hydroxyglutaric aciduria (MIM#615182) (Nota et al 2013). Citrate is central to TCA cycle and it caries acetyl-units generated by pyruvate dehydrogenase or fatty acid oxidation from the mitochondrial matrix into the cytosol to supply acetyl-CoA to de novo lipogenesis, cholesterol synthesis (Fig. 8a, b) and histone acetylation (Wellen et al 2009). Control and patient fibroblasts were incubated with M_5_ [U-^13^C]-glutamine. Conversion of the carbon skeleton of glutamine into citrate proceeds partly in the mitochondrion and partly in the cytosol via successive deamidation and deamination of glutamine to 2-oxoglutarate in mitochondria and reductive carboxylation of 2-oxoglutarate to isocitrate catalyzed by cytosolic isocitrate dehydrogenase 1 (Mullen et al 2011) (Metallo et al 2011). In control fibroblasts, a prominent fractional contribution of M_4_ citrate to the MID of citrate was observed. Mitochondrial M_5_ 2-oxoglutarate from M_5_ [U-^13^C]-glutamine is converted into M_4_-oxaloacetate by the TCA cycle. Furthermore, cytosolic M_5_ citrate from M_5_ [U-^13^C]-glutamine enters the mitochondria and is also converted into M_4_ oxaloacetate (Fig. 8b). Very little M_5_ citrate was observed in control fibroblasts. In patient fibroblasts, however, a high fractional contribution of M_5_ citrate to the MID of citrate was observed, confirming the severely impaired entrance of cytosolic citrate into mitochondria in patient fibroblast for further oxidation.

Next, the fate of the label was followed into cholesterol, a kind of ‘13/14-polymer’ of acetate (Hellerstein and Neese 1992; Kelleher et al 1994). Acetate used in the cholesterol synthesis comes partially from citrate. Labeling of citrate by acetate coming from [U-^13^C]-glucose takes place inside mitochondria and labeled citrate needs to be exported to provide acetate for the cholesterol biosynthesis (Fig. 8a). When control fibroblasts were incubated with [U-^13^C]-glucose the fractional contribution of M_6_–M_10_ mass isotopomers dominated the MID of cholesterol, indicative of considerable labeling of acetate pool. In patient fibroblasts a shift was observed to lower mass isotopomers of cholesterol indicative of less efficient labeling of acetate. The transport of mitochondrial labeled citrate to the cytosol was severely impaired when SLC25A1 is deficient.

Synthesis of citrate from glutamine takes place in the cytosol by reductive carboxylation of 2-ketoglutarate (Fig. 8b). M_5_ [U-^13^C]-glutamine gives rise to M_5_ citrate in the cytosol, which is split into M_3_ oxaloacetate and M_2_ acetyl-CoA by ATP-citrate lyase. When control fibroblasts were incubated with [U-^13^C]-glutamine a minor contribution of M_2_ cholesterol was observed. Apparently, in control fibroblasts glutamine-derived acetyl-CoA is of very limited importance in the supply of acetate to cholesterol biosynthesis. In patient fibroblast, however, the picture was quite different with an increased fraction of M_4_ cholesterol, indicative for a more prominent labeling of acetyl-CoA by [U-^13^C]-glutamine. In the absence of the mitochondrial citrate transporter glutamine became a donor of acetate-units to cholesterol synthesis.

The results of this study are very similar to those obtained in the lung tumor cell line H460 (Jiang et al 2016). SLC25A1 has been implicated in tumor progression (Kolukula et al 2014, Catalina-Rodriguez et al 2012). Among other things, Jiang et al studied the transfer of ^13^C from [U-^13^C]-glucose and [U-^13^C]-glutamine to palmitate, the ‘octamer’ of the monomer acetate. After incubation of H460 cells with [U-^13^C]-glucose palmitate MID was dominated by M_10_–M_16_ mass isotopomers, indicative of a high flux of M_2_ citrate from the mitochondria into the cytosol resulting in a very efficient labeling of the cytosolic acetate pool. Deletion of SLC25A1 by CRISPR/Cas9 shifted the fractional contribution of the dominant mass isotopomers of the MID of palmitate to lower mass isotopomers (M_2_–M_8_), reflecting a restricted efflux of M_2_ citrate from mitochondria into the cytosol with a lower efficiency of labeling of the acetate pool for palmitate biosynthesis. When H460 cells were incubated with [U-^13^C]-glutamine, only M_2_–M_4_ mass isotopomers were observed in the MID of palmitate. After deletion of SLC25A1 labeling efficiency of acetate by [U-^13^C]-glutamine increased, with M_6_–M_10_ as the dominant mass isotopomers of palmitate.

## Conclusions and future perspectives

In Table 1 an overview is given of the major results and possible therapeutic consequences of the studies on metabolic reprogramming in patients with an IEM and related model systems with stable isotopes. The summary clearly shows that whole body metabolism adapts in a way which was quite unexpected and which was very specific for each of the IEMs individually. It clearly points to the need of in vivo studies both in patients and in animal models of IEM.

**Table 1:** Summary of results of flux analysis in IEM

IEM	Results
GSD Ia	- Appreciable postprandial glucose production - Glucose production is due to glucosidase activity in liver - Intracellular de novo lipid metabolism is strongly enhanced - Lipoprotein metabolism seems to be impaired with reduced lipolysis - Metabolic reprogramming is driven by the transcription factor ChREBP activated by G6P
MCADD	- Normal oxidation of medium-chain fatty acids. - The hypoglycemia is driven by a persistent increased consumption of glucose by peripheral tissue and an inadequate production of glucose by gluconeogenesis - Supply of gluconeogenic substrates by peripheral tissue might be too low. - A protein-enriched diet might be an additional therapeutic option for patient with MCADD to boost gluconeogenesis to prevent a hypoglycemic hypoketotic crisis. - There might be an additional role of peroxisomes and endoplasmic reticulum to fatty acid oxidation
PMA	- Propionate oxidation is almost higher in patients with PMA than in healthy volunteers - Gut microbiota are an important producer of propionate - A modified β oxidation pathway of propionate exists in humans, with cytotoxic intermediates - Changing the microbiota composition by dietary intervention might be a therapeutic option
UCD	- First pass absorption by the liver of ammonia from the gut is ~70% and 30% enters the systemic circulation. - Direct conversion of ammonia into urea upon first pass is limited to 35% of the supplied ammonia. - Remainder of supplied ammonia is used in the synthesis of glutamine, in liver and in muscle - Glutamine is central in whole body nitrogen metabolism and in urea synthesis - 2-Ketoglutarate supply in hepatic mitochondria is an important precursor in whole body glutamate and glutamine synthesis - Additional gifts of glutamate to enhance glutamine synthesis during hyperammonemic events might be an additional therapeutic option - Re-interpretation of changes in glutamine concentration in blood during the monitoring of therapy might be necessary
PKU	- There is an as yet unidentified pathway of phenylalanine oxidation.
D,L-2OHG-uria	- Preliminary results in fibroblast point to a shift from glucose to glutamine to support lipid and cholesterol synthesis

Besides studying reprogrammed metabolism, it also became clear that the study of the role of gut microbiota in the clinical presentation of IEMs urgently needs closer examination. In mFAO the rate of microbial synthesis of products like arylcarboxylates, in PA and MMA of SCFA, and in UCDs of ammonia might be of importance to understand the broad spectrum of clinical presentation of IEMs. There are ample studies showing that both human genetic variation and diet have clear effects on the composition of the gut microbiota, predisposing to microbiome dysbiosis (see (Hall et al 2017) for a review). It can be expected that also in the case of IEM, microbiome composition is altered and possibly leads to microbiome dysbiosis.

In only a limited set of IEM extensive studies have been performed. More model systems are required to address the many issues in the pathology of IEMs. Genetically modified mice are the main model system, but they have their drawbacks. A clear example is the GALT knockout mice model for galactosemia, which did not show the clinical phenotype seen in children suffering from galactosemia (Leslie et al 1996). The use of zebrafish as an alternative to mice is an emergent field (Wager et al 2014). New cell models and methods offer new opportunities for the study of IEM (Wangler et al 2017).

Great progress has been made in the development of induced specific cell types starting from patient fibroblasts (Xu et al 2015). New methods in tracer-based metabolomics allows for fingerprinting of metabolism with unsupervised non-targeted tracer fate detection (Hiller et al 2013; Weindl et al 2016). Mathematical models are continuously being improved to convert the observed MIDs of metabolites into flux pattern (for reviews see (Buescher et al 2015; Vasilakou et al 2016)).

In tumors, metabolism is also forced to reprogram itself because of mutations in genes coding for proteins involved in cellular growth and associated metabolism. Sometimes, enzyme deficiencies known in the field of IEMs results in tumor formation, showing the overlap of both fields with respect to the many ways metabolism can reorganize itself (see (Erez and DeBerardinis 2015; DeBerardinis and Thompson 2012) for reviews). Many metabolic studies in tumors testify to the extreme versatility of metabolism to sustain maintenance and growth of cells (see (Wong et al 2017) for a review).

Systems biology might offer a new way to integrate multi-omics data into molecular networks (Argmann et al 2016). Recently, a community-driven global reconstruction of human metabolism resulted in the comprehensive genome-scale metabolic map Recon 2 (Thiele et al 2013). This metabolic map has already been used to predict additional metabolic biomarkers of IEMs (Sahoo et al 2012; Thiele et al 2013; Shlomi et al 2009). One might use this map not only to predict biomarkers but also to diagnose as yet unknown inborn errors of metabolism by projecting abnormal metabolite profiles and genome-wide exome sequence data onto this map. This is now actively pursued in the Undiagnosed Diseases Network in the USA and internationally (Taruscio et al 2015; Ramoni et al 2017; Austin et al 2017). The map can also be used to elucidate affected pathways in known inborn errors of metabolism. In this way the targeted approach applied for instance in the study of lysine metabolism (Struys and Jakobs 2010; Posset et al 2014) to elucidate defective metabolic pathways, can now be transformed into a genomic-wide approach. Very recently, a systems biology approach has been undertaken to integrate Recon-2 with metabolomics data of the NCI-60 panel of 60 well characterized primary human cancer cell lines established from nine common tumor types (Jain et al 2012, Aurich et al 2017). The results of extracellular metabolomics reflecting uptake and release of metabolites were used to generate condition- and cell-specific metabolic models of the cancer cell lines. Next, these metabolic models were analyzed for their capability to harvest energy and generate cofactors. This analysis revealed the different strategies cells can use to cope with environmental and genetic perturbations. Stable isotope based metabolomics can then be used to test the hypotheses generated. This looks a very promising way to study metabolic reprogramming of patient fibroblasts or fibroblast-derived induced specific cell types.

Textbook knowledge is clearly insufficient to deduce the way metabolism copes with an enzyme deficiency. Metabolism and its regulation appear to be too versatile. Methods are now available to generate hypotheses about the flux distribution in the case of IEMs on a genome-wide scale and cell specific. Stable isotopes methodology is the tool to test these hypotheses. Hopefully, these new technologies will help us to get a better understanding of how metabolism is reprogrammed in patients with an IEM such that we are able to offer these patients better treatments for a better life.

## References

[CR1] Ando T, Nyhan WL, Connor JD (1972). The oxidation of glycine and propionic acid in propionic acidemia with ketotic hyperglycinemia. Pediatr Res.

[CR2] Ando T, Rasmussen K, Nyhan WL, Hull D (1972). 3-hydroxypropionate: significance of -oxidation of propionate in patients with propionic acidemia and methylmalonic acidemia. Proc Natl Acad Sci U S A.

[CR3] Argmann CA, Houten SM, Zhu J, Schadt EE (2016). A next generation multiscale view of inborn errors of metabolism. Cell Metab.

[CR4] Aurich MK, Fleming RMT, Thiele I (2017). A systems approach reveals distinct metabolic strategies among the NCI-60 cancer cell lines. PLoS Comput Biol.

[CR5] Austin CP, Cutillo CM, Lau LPL et al (2017) Future of rare diseases research 2017-2027: an IRDiRC perspective. Clin Transl Sci. 10.1111/cts.12500 10.1111/cts.12500 PMC5759721 28796445

[CR6] Bandsma RH, Wiegman CH, Herling AW (2001). Acute inhibition of glucose-6-phosphate translocator activity leads to increased de novo lipogenesis and development of hepatic steatosis without affecting VLDL production in rats. Diabetes.

[CR7] Bandsma RHJ, Prinsen BH, de Sain-van d, Velden M (2008). Increased de novo Lipogenesis and delayed conversion of large VLDL into intermediate density lipoprotein particles contribute to Hyperlipidemia in glycogen storage disease type 1a. Pediatr Res.

[CR8] Bandsma RHJ, Rake J-P, Visser G (2002). Increased lipogenesis and resistance of lipoproteins to oxidative modification in two patients with glycogen storage disease type 1a. J Pediatr.

[CR9] Besten den G, Havinga R, Bleeker A (2014). The short-chain fatty acid uptake fluxes by mice on a guar gum supplemented diet associate with amelioration of major biomarkers of the metabolic syndrome. PLoS One.

[CR10] Bian F, Kasumov T, Thomas KR (2005). Peroxisomal and mitochondrial oxidation of fatty acids in the heart, assessed from the 13C labeling of malonyl-CoA and the acetyl moiety of citrate. J Biol Chem.

[CR11] Bonnefont J-P, Bastin J, Behin A, Djouadi F (2009). Bezafibrate for an inborn mitochondrial beta-oxidation defect. N Engl J Med.

[CR12] Bougneres PF, Balasse EO, Ferre P, Bier DM (1986). Determination of ketone body kinetics using a D-(−)-3-hydroxy[4,4,4-2H3]butyrate tracer. J Lipid Res.

[CR13] Bougneres PF, Ferre P (1987). Study of ketone body kinetics in children by a combined perfusion of 13C and 2H3 tracers. Am J Phys.

[CR14] Brosnan JT, Brosnan ME, Charron R, Nissim I (1996). A mass isotopomer study of urea and glutamine synthesis from 15N-labeled ammonia in the perfused rat liver. J Biol Chem.

[CR15] Brosnan JT, Brosnan ME, Nissim I (2004). A window into cellular metabolism: hepatic metabolism of (15)N-labelled substrates. Metab Eng.

[CR16] Buescher JM, Antoniewicz MR, Boros LG (2015). A roadmap for interpreting (13)C metabolite labeling patterns from cells. Curr Opin Biotechnol.

[CR17] Catalina-Rodriguez O, Kolukula VK, Tomita Y (2012). The mitochondrial citrate transporter, CIC, is essential for mitochondrial homeostasis. Oncotarget.

[CR18] Clayton TA (2012). Metabolic differences underlying two distinct rat urinary phenotypes, a suggested role for gut microbial metabolism of phenylalanine and a possible connection to autism. FEBS Lett.

[CR19] DeBerardinis RJ, Thompson CB (2012). Cellular metabolism and disease: what do metabolic outliers teach us?. Cell.

[CR20] Derks TGJ, Boer TS, van Assen A (2008). Neonatal screening for medium-chain acyl-CoA dehydrogenase (MCAD) deficiency in The Netherlands: the importance of enzyme analysis to ascertain true MCAD deficiency. J Inherit Metab Dis.

[CR21] Derks TGJ, van Dijk TH, Grefhorst A (2008). Inhibition of mitochondrial fatty acid oxidation in vivo only slightly suppresses gluconeogenesis but enhances clearance of glucose in mice. Hepatology.

[CR22] Erez A, DeBerardinis RJ (2015). Metabolic dysregulation in monogenic disorders and cancer-finding method in madness. Nat Rev Cancer.

[CR23] Fletcher JM, Pitt JJ (2001). Fasting medium chain acyl-coenzyme a dehydrogenase--deficient children can make ketones. Metab Clin Exp.

[CR24] Gobin-Limballe S, Djouadi F, Aubey F (2007). Genetic basis for correction of very-long-chain acyl-coenzyme a dehydrogenase deficiency by bezafibrate in patient fibroblasts: toward a genotype-based therapy. Am J Hum Genet.

[CR25] Grefhorst A, Schreurs M, Oosterveer MH (2010). Carbohydrate-response-element-binding protein (ChREBP) and not the liver X receptor α (LXRα) mediates elevated hepatic lipogenic gene expression in a mouse model of glycogen storage disease type 1. Biochem J.

[CR26] Hakvoort TBM, He Y, Kulik W (2017). Pivotal role of glutamine Synthetase in ammonia detoxification. Hepatology.

[CR27] Hall AB, Tolonen AC, Xavier RJ (2017) Human genetic variation and the gut microbiome in disease. Nat Rev Genet 18:690–699. doi: 10.1038/nrg.2017.63 10.1038/nrg.2017.63 28824167

[CR28] He Y, Hakvoort TBM, Kohler SE (2010). Glutamine Synthetase in muscle is required for glutamine production during fasting and Extrahepatic ammonia detoxification. J Biol Chem.

[CR29] Heales SJ, Thompson GN, Massoud AF (1994). Production and disposal of medium-chain fatty acids in children with medium-chain Acyl-CoA Dehydrogenase deficiency. J Inherit Metab Dis.

[CR30] Hellerstein MK, Neese RA (1992). Mass isotopomer distribution analysis: a technique for measuring biosynthesis and turnover of polymers. Am J Phys.

[CR31] Hellerstein MK, Neese RA, Linfoot P (1997). Hepatic gluconeogenic fluxes and glycogen turnover during fasting in humans. A stable isotope study. J Clin Invest.

[CR32] Herrema H, Derks TGJ, van Dijk TH (2008). Disturbed hepatic carbohydrate management during high metabolic demand in medium-chain acyl-CoA dehydrogenase (MCAD)-deficient mice. Hepatology.

[CR33] Hijmans BS, Boss A, van Dijk TH (2017). Hepatocytes contribute to residual glucose production in a mouse model for glycogen storage disease type Ia. Hepatology.

[CR34] Hiller K, Wegner A, Weindl D (2013). NTFD--a stand-alone application for the non-targeted detection of stable isotope-labeled compounds in GC/MS data. Bioinformatics.

[CR35] Houten SM, Herrema H, Brinke Te H (2013). Impaired amino acid metabolism contributes to fasting-induced hypoglycemia in fatty acid oxidation defects. Hum Mol Genet.

[CR36] Huidekoper HH, Visser G, Ackermans MT (2010). A potential role for muscle in glucose homeostasis: in vivo kinetic studies in glycogen storage disease type 1a and fructose-1,6-bisphosphatase deficiency. J Inherit Metab Dis.

[CR37] Jain M, Nilsson R, SS S (2012). Metabolite profiling indentifies a key role for Glycine in rapid cancer cell proliferation. Science.

[CR38] Jiang L, Boufersaoui A, Yang C (2016). Metabolic engineering. Metab Eng.

[CR39] Jiang S, Wells CD, Roach PJ (2011). Starch-binding domain-containing protein 1 (Stbd1) and glycogen metabolism: identification of the Atg8 family interacting motif (AIM) in Stbd1 required for interaction wirg GABARAPL1. Biochem Biophys Res Commun.

[CR40] Jin Z, Bian F, Tomcik K (2015). Compartmentation of metabolism of the C_12_-, C_9_-, and C_5_-*n*-dicarboxylates in rat liver, investigated by mass Isotopomer analysis. J Biol Chem.

[CR41] Jones JG, Garcia P, Barosa C (2009). Hepatic anaplerotic outflow fluxes are redirected from gluconeogenesis to lactate synthesis in patients with type 1a glycogen storage disease. Metab Eng.

[CR42] Kalderon B, Korman SH, Gutman A, Lapidot A (1989). Estimation of glucose carbon recycling in children with glycogen storage disease: a ^13^CNMR study using [U-^13^C] glucose. Proc Natl Acad Sci U S A.

[CR43] Kalderon B, Korman SH, Gutman A, Lapidot A (1989). Glucose recycling and production in glycogenosis type Iand III: stable isotope technique study. Am J Physiol Endocrinol Metab.

[CR44] Kalderon B, Lapidot A, Korman SH, Gutman A (1988). Glucose recycling and production in children with glycogen storage disease type I, studied by gas chromatography/mass spectrometry and (U-13C)glucose. Biomed Environ Mass Spectrom.

[CR45] Kalivianakis M, Verkade HJ, Stellaard F (1997). The 13C-mixed triglyceride breath test in healthy adults: determinants of the 13CO2 response. Eur J Clin Investig.

[CR46] Kasumov T, Adams JE, Bian F (2005). Probing peroxisomal beta-oxidation and the labelling of acetyl-CoA proxies with [1-(13C)]octanoate and [3-(13C)]octanoate in the perfused rat liver. Biochem J.

[CR47] Kelleher JK, Kharroubi AT, Aldaghlas TA (1994). Isotopomer spectral analysis of cholesterol synthesis: applications in human hepatoma cells. Am J Phys.

[CR48] Kelleher JK, Masterson TM (1992). Model equations for condensation biosynthesis using stable isotopes and radioisotopes. Am J Phys.

[CR49] Kelleher JK, Nickol GB (2015) Isotopomer spectral analysis: utilizing nonlinear models in isotopic flux studies. Methods Enzymol 561:303-30. doi: 10.1016/bs.mie.2015.06.039. 10.1016/bs.mie.2015.06.039 26358909

[CR50] Kolukula VK, Sahu G, Wellstein A (2014). SLC25A1, or CIC, is a novel transcriptional target of mutant p53 and a negative tumor prognostic marker. Oncotarget.

[CR51] Kormanik K, Kang H, Cuebas D (2012). Evidence for involvement of medium chain acyl-CoA dehydrogenase in the metabolism of phenylbutyrate. Mol Genet Metab.

[CR52] Lee B, Hong Y, Jahoor F (2000). In vivo urea cycle flux distinguishes and correlates with phenotypic severity in disorders of the urea cycle. Proc Natl Acad Sci U S A.

[CR53] Lei KJ, Chen H, Pan CJ (1996). Glucose-6-phosphatase dependent substrate transport in the glycogen storage disease type-1a mouse. Nat Genet.

[CR54] Lenzen S (2014) A fresh view of glycolysis and glucokinase regulation: history and current status. J Biol Chem 289:12189–12194. 10.1074/jbc.R114.557314 10.1074/jbc.R114.557314 PMC4007419 24637025

[CR55] Leslie ND, Yager KL, McNamara PD, Segal S (1996) A mouse model of galactose-1-phosphate Uridyl Transferase deficiency. Biochem Mol Med 59:7–12 10.1006/bmme.1996.0057 8902187

[CR56] Marini JC, Lee B, Garlick PJ (2006a) Reduced ornithine transcarbamylase activity does not impair ureagenesis in *Otc*^spf-ash^ mice. J Nutr 136:1017–1020 10.1093/jn/136.4.1017 16549467

[CR57] Marini JC, Lee B, Garlick PJ (2006b) Ornithine restores ureagenesis capacity and mitigates hyperammonemia in *Otc*^spf-ash^ mice. J Nutr 136:1834–1838 10.1093/jn/136.7.1834 16772445

[CR58] Martines A-CMF, van Eunen K, Reijngoud D-J, Bakker BM (2017). The promiscuous enzyme medium-chain 3-keto-acyl-CoA thiolase triggers a vicious cycle in fatty-acid beta-oxidation. PLoS Comput Biol.

[CR59] Matini JC, Lee B, Garlick PJ (2005). In vivo urea kinetics studies in conscious mice. J Nutr.

[CR60] Matthews DE, Downey RS (1984). Measurement of urea kinetics in humans: a validation of stable isotope tracer methods. Am J Phys.

[CR61] Meija J, Coplen TB, Berglund M (2016). Isotopic compositions of the elements 2013 IUPAC technical report. Pure Appl Chem.

[CR62] Metallo CM, Gameiro PA, Bell EL (2011). Reductive glutamine metabolism by IDH1 mediates lipogenesis under hypoxia. Nature.

[CR63] Midani FS, Wynn ML, Schnell S (2017). The importance of accurately correcting for the natural abundance of stable isotopes. Anal Biochem.

[CR64] Mullen AR, Wheaton WW, Jin ES (2011). Reductive carboxylation supports growth in tumour cells with defective mitochondria. Nature.

[CR65] Mutel E, Abdul-Wahed A, Ramamonjisoa N (2011). Targeted deletion of liver glucose-6 phosphatase mimics glycogen storage disease type 1a including development of multiple adenomas. J Hepatol.

[CR66] Nissim I, Brosnan ME, Yudkoff M, Brosnan JT (1999). Studies of hepatic glutamine metabolism in the perfused rat liver with (15)N-labeled glutamine. J Biol Chem.

[CR67] Nissim I, Cattano C, Nissim I, Yudkoff M (1992) Relative role of the glutaminase, glutamate dehydrogenase, and AMP-deaminase pathways in hepatic ureagenesis: studies with 15N. Arch Biochem Biophys 292:393–401 10.1016/0003-9861(92)90008-k 1346240

[CR68] Nissim I, Horyn O, Luhovyy B (2003). Role of the glutamate dehydrogenase reaction in furnishing aspartate nitrogen for urea synthesis: studies in perfused rat liver with 15N. Biochem J.

[CR69] Nissim I, Yudkoff M, Brosnan JT (1996). Regulation of [15N]urea synthesis from [5-15N]glutamine. Role of pH, hormones, and pyruvate. J Biol Chem.

[CR70] Nota B, Struys EA, Pop A (2013). Deficiency in SLC25A1, encoding the mitochondrial citrate carrier, causes combined D-2- and L-2-hydroxyglutaric aciduria. Am J Hum Genet.

[CR71] Oosterveer MH, Grefhorst A, van Dijk TH (2009). Fenofibrate simultaneously induces hepatic fatty acid oxidation, synthesis, and elongation in mice. J Biol Chem.

[CR72] Posset R, Opp S, Struys EA (2014). Understanding cerebral L-lysine metabolism: the role of L-pipecolate metabolism in Gcdh-deficient mice as a model for glutaric aciduria type I. J Inherit Metab Dis.

[CR73] Powell RC, Wentworth SM, Brandt IK (1981). Endogenous glucose production in type I glycogen storage disease. Metabolism.

[CR74] Ramoni RB, Mulvihill JJ, Adams DR (2017). The undiagnosed diseases network: accelerating discovery about health and disease. Am J Hum Genet.

[CR75] Reijngoud D-J, Verkade HJ, Schierbeek H (2017). Applications in fat absorption and metabolism. Mass spectrometry and stable isotopes in nutritional and pediatric research.

[CR76] Reszko AE, Kasumov T, David F (2004). Peroxisomal fatty acid oxidation is a substantial source of the acetyl moiety of malonyl-CoA in rat heart. J Biol Chem.

[CR77] Richards P, Ourabah S, Montagne J (2017). MondoA/ChREBP: the usual suspects of transcriptional glucose sensing; implication in pathophysiology. Metab Clin Exp.

[CR78] Rinaldo P, O'Shea JJ, Welch RD, Tanaka K (1990). The enzymatic basis for the dehydrogenation of 3-phenylpropionic acid: in vitro reaction of 3-phenylpropionyl-CoA with various acyl-CoA dehydrogenases. Pediatr Res.

[CR79] Rother KI, Schwenk F (1995). Glucose production in glycogen storage disease I is not associated with increased cycling through hepatic glycogen. Am J Physiol Endocrinol Metab.

[CR80] Sahoo S, Franzson L, Jonsson JJ, Thiele I (2012). A compendium of inborn errors of metabolism mapped onto the human metabolic network. Mol BioSyst.

[CR81] Scaglia F, Marini JC, Rosenberger J (2003). Differential utilization of systemic and enteral ammonia for urea synthesis in control subjects and ornithine transcarbamylase deficiency carriers. Am J Clin Nutr.

[CR82] Scaglia F, Zheng Q, O'Brien WE (2002). An integrated approach to the diagnosis and prospective management of partial ornithine transcarbamylase deficiency. Pediatrics.

[CR83] Shlomi T, Cabili MN, Ruppin E (2009) Predicting metabolic biomarkers of human inborn errors of metabolism. Mol Syst Biol. 5:263. doi: 10.1038/msb.2009.22 10.1038/msb.2009.22 PMC2683725 19401675

[CR84] Struys EA, Jakobs C (2010). Metabolism of lysine in aminoadipic semialdehyde dehydrogenasedeficient fibroblasts: evidence for an alternative pathway of pipecolic acid formation. FEBS Lett.

[CR85] Taruscio D, Groft SC, Cederroth H (2015). Undiagnosed disease network international (UDNI): white paper for global actions to meet patient needs. Mol Genet Metab.

[CR86] Thiele I, Swainston N, Fleming RMT (2013). A community-driven global reconstruction of human metabolism. Nat Biotechnol.

[CR87] Thompson GN, Halliday D (1990). Significant phenylalanine hydroxylation in vivo in patients with classical phenylketonuria. J Clin Invest.

[CR88] Thompson GN, Walter JH, Bresson JL (1990). Sources of propionate in inborn errors of propionate metabolism. Metab Clin Exp.

[CR89] Thompson GN, Walter JH, Leonard JV, Halliday D (1990). In vivo enzyme activity in inborn errors of metabolism. Metabolism.

[CR90] Tolwani RJ, Hamm DA, Tian L (2005). Medium-chain acyl-CoA dehydrogenase deficiency in gene-targeted mice. PLoS Genet.

[CR91] Tredwell GD, Keun HC (2015). convISA: a simple, convoluted method for isotopomer spectral analysis of fatty acids and cholesterol. Metab Eng.

[CR92] Tsalikian E, Simmons P, Gerich JE (1984). Glucose production and utilization in childrenwith glycogen storage disease type I. Am J Physiol Endocrinol Metab.

[CR93] van Dijk TH, van der Sluijs FH, Wiegman CH (2001). Acute inhibition of hepatic glucose-6-phosphatase does not affect gluconeogenesis but directs gluconeogenic flux toward glycogen in fasted rats. A pharmacological study with the chlorogenic acid derivative S4048. J Biol Chem.

[CR94] van Eunen K, Simons SMJ, Gerding A (2013). Biochemical competition makes fatty-acid β-oxidation vulnerable to substrate overload. PLoS Comput Biol.

[CR95] van Eunen K, Volker-Touw CML, Gerding A (2016). Living on the edge: substrate competition explains loss of robustness in mitochondrial fatty-acid oxidation disorders. BMC Biol.

[CR96] van Spronsen FJ, Reijngoud DJ, Smit GP (1998). Phenylketonuria. The in vivo hydroxylation rate of phenylalanine into tyrosine is decreased. J Clin Invest.

[CR97] Vasilakou E, Machado D, Theorell A (2016). Current state and challenges for dynamic metabolic modeling. Curr Opin Microbiol.

[CR98] Veeneman JM, Kingma HA, Stellaard F (2004). Comparison of amino acid oxidation and urea metabolism in haemodialysis patients during fasting and meal intake. Nephrology Dialysis Transplantation.

[CR99] Veeneman JM, Kingma HA, Stellaard F (2005). Oxidative metabolism appears to be reduced in long-term hemodialysis patients. Am J Kidney Dis.

[CR100] Violante S, Ijlst L, Brinke Te H (2013). Peroxisomes contribute to the acylcarnitine production when the carnitine shuttle is deficient. Biochim Biophys Acta.

[CR101] Wager K, Mahmood F, Russell C (2014). Modelling inborn errors of metabolism in zebrafish. J Inherit Metab Dis.

[CR102] Wanders RJA (2014). Metabolic functions of peroxisomes in health and disease. Biochimie.

[CR103] Wanders RJA, Komen J, Kemp S (2010). Fatty acid omega-oxidation as a rescue pathway for fatty acid oxidation disorders in humans. FEBS J.

[CR104] Wanders RJA, Waterham HR, Ferdinandusse S (2016) Metabolic interplay between peroxisomes and other subcellular organelles including mitochondria and the endoplasmic reticulum. Front Cell Dev Biol 3:1752. 10.1016/0005-2736(87)90321-X 10.3389/fcell.2015.00083 PMC4729952 26858947

[CR105] Wangler MF, Yamamoto S, Chao H-T (2017). Model organisms facilitate rare disease diagnosis and therapeutic research. Genetics.

[CR106] Watson E, Olin-Sandoval V, Hoy MJ et al (2016) Metabolic network rewiring of propionate flux compensates vitamin B12 deficiency in C. Elegans. elife. 10.7554/eLife.17670 10.7554/eLife.17670 PMC4951191 27383050

[CR107] Weghuber D, Mandl M, Krssak M (2007). Characterization of hepatic and brain metabolism in young adults with glycogen storage disease type 1: a magnetic resonance spectroscopy study. Am J Physiol Endocrinol Metab.

[CR108] Weindl D, Wegner A, Hiller K (2016). MIA: non-targeted mass isotopolome analysis. Bioinformatics.

[CR109] Wellen KE, Hatzivassiliou G, Sachdeva UM et al (2009) ATP-citrate lyase links cellular metabolism to histone acetylation. Science 324:1076–1080 10.1126/science.1164097 PMC2746744 19461003

[CR110] Wilson KA, Han Y, Zhang M et al (2017) Interrelations between 3-hydroxypropionate and propionate metabolism in rat liver: relevance to disorders of propionyl-CoA metabolism. Am J Physiol Endocrinol Metab 10.1152/ajpendo.00105.2017 10.1152/ajpendo.00105.2017 PMC5668600 28634175

[CR111] Wong TL, Che N, Ma S (2017). Reprogramming of central carbon metabolism in cancer stem cells. Biochim Biophys Acta.

[CR112] Xu J, Du Y, Deng H (2015). Direct lineage reprogramming: strategies, mechanisms, and applications. Cell Stem Cell.

[CR113] Yang D, Hazey JW, David F (2000). Integrative physiology of splanchnic glutamine and ammonium metabolism. Am J Physiol Endocrinol Metab.

[CR114] Yudkoff M, Daikhin Y, Ye X (1998). In vivo measurement of ureagenesis with stable isotopes. J Inherit Metab Dis.

